# Inference and multiscale model of epithelial-to-mesenchymal transition via single-cell transcriptomic data

**DOI:** 10.1093/nar/gkaa725

**Published:** 2020-09-01

**Authors:** Yutong Sha, Shuxiong Wang, Peijie Zhou, Qing Nie

**Affiliations:** Department of Mathematics, University of California, Irvine, Irvine, CA 92697, USA; The NSF-Simons Center for Multiscale Cell Fate Research, University of California, Irvine, Irvine, CA 92697, USA; Department of Mathematics, University of California, Irvine, Irvine, CA 92697, USA; Department of Mathematics, University of California, Irvine, Irvine, CA 92697, USA; Department of Mathematics, University of California, Irvine, Irvine, CA 92697, USA; The NSF-Simons Center for Multiscale Cell Fate Research, University of California, Irvine, Irvine, CA 92697, USA; Department of Developmental and Cell Biology, University of California, Irvine, Irvine, CA 92697, USA

## Abstract

Rapid growth of single-cell transcriptomic data provides unprecedented opportunities for close scrutinizing of dynamical cellular processes. Through investigating epithelial-to-mesenchymal transition (EMT), we develop an integrative tool that combines unsupervised learning of single-cell transcriptomic data and multiscale mathematical modeling to analyze transitions during cell fate decision. Our approach allows identification of individual cells making transition between all cell states, and inference of genes that drive transitions. Multiscale extractions of single-cell scale outputs naturally reveal intermediate cell states (ICS) and ICS-regulated transition trajectories, producing emergent population-scale models to be explored for design principles. Testing on the newly designed single-cell gene regulatory network model and applying to twelve published single-cell EMT datasets in cancer and embryogenesis, we uncover the roles of ICS on adaptation, noise attenuation, and transition efficiency in EMT, and reveal their trade-off relations. Overall, our unsupervised learning method is applicable to general single-cell transcriptomic datasets, and our integrative approach at single-cell resolution may be adopted for other cell fate transition systems beyond EMT.

## INTRODUCTION

The epithelial-to-mesenchymal transition (EMT) is an important process observed in many biological systems, including embryogenesis, wound healing and malignant progression (1). Recently, several lines of *in vitro* and *in vivo* evidence, along with computational modeling, suggest that cells undergoing EMT is not a simple binary switch, and during the transition some cells exhibit mixed features of both epithelial and mesenchymal features (1,2). Those cells characterized as intermediate cell state (ICS) have been implicated in the potential roles of stemness, collective migration, drug resistance, metastasis, and noise control (1,3,4).

Key gene regulatory elements of EMT, such as EMT-suppressing microRNAs and EMT-promoting transcriptional factors, have been used for modeling and experimental analysis of ICS. Existence of multi-stable states of the modeled gene regulatory networks has been used to imply existence of ICS (5–7). Few regulators have been found to be critical in formation of ICS, such as a transcriptional factor Ovol for regulating growth and Notch signaling for cell-cell communications (7–9), and few others have been suggested in stabilizing ICS (10–12).

What are the functional advantages of ICS in state transitions? Cell population modeling suggests the increased number of ICS attenuates the fluctuations in cell numbers during transition (13) in addition to help maintain the mean of signal response (14). Experimental and modeling analysis shows ICS can also facilitate the robustness of population dynamics (15). Signal adaptation has been found to tightly constrain gene regulations (16), and however, could be important as a ‘survival strategy’ in growth and migration of cells (17). At the level of gene regulations, achieving robustness and signal adaptation, which both are important to cell fate transition, often require different, sometimes competitive, gene regulations (18). Comparisons of ICS across different EMT systems remain a major open problem (19).

Are the cells in ICS showing strong variability or tightly controlled? Single-cell RNA sequencing (scRNA-seq) technology provides unprecedented opportunities to explore cellular heterogeneity, distinct cell states, marker genes and the accompanying functions (20–22). Expression levels of epithelial and mesenchymal markers and transcription factors of ICS have been recently analyzed in EMT at single-cell resolution (23). EMT scoring metrics have been developed by applying the best-fit model obtained from a previously-developed iterative statistical procedure to quantify EMT status of cells in different cell lines (24–26). More recently, a topographic map underlying EMT has been constructed to explore ICS for its phenotypic plasticity (27).

One major challenge is to analyze temporal dynamics of cells in EMT from the snapshot transcriptomic data. Pseudo-temporal ordering (pseudotime) of cells in scRNA-seq data provides trajectories of cells that may recapitulate transition between cell states. However, such approach is usually dependent on the cell-embedding in the low-dimensional space via dimension reduction or structured graphs (28–30). Recently, the single-cell method SOUP allows classification of both pure and intermediate cells by constructing the cell-cell similarity matrix and estimating a membership matrix (28). Robust tools to quantify the transition trajectories and detect driving genes in EMT are still in need.

What are the transitional properties of cells near or at ICS? Is ICS simply another stable cell state between epithelial and mesenchymal states? Can we construct and quantify the transition paths in EMT? Here, we first develop an unsupervised learning method (QuanTC) to infer and quantify transitional property of individual cells in scRNA-seq data. After validating against our EMT multiscale single-cell model, which combines several previously published gene regulatory networks, we apply QuanTC to twelve published EMT transcriptomic datasets in cancer and embryogenesis. By inspecting transition cells, ICS, and their relationship with epithelial and mesenchymal states, we construct the ICS-regulated EMT trajectories. We then compare the inferred transition trajectories, which are different between cancer and embryogenesis, with another method based on critical transition theory, and re-construct core gene regulatory circuits for the published datasets to analyze the similarity and consistency in state transition.

To further investigate the inferred trajectories shared by various EMT systems, we develop and analyze cell transition models by defining and measuring three metrics emergent from EMT cell population dynamics. Differences between inferred EMT trajectories and their integrations with scRNA-seq data are then analyzed. Our integrative approach, which fuses unsupervised learning of gene expression data at single-cell resolution along with principle-guided cell population model, provides multiscale effective connections between genes and cells in analyzing complex cell fate decision that involves ICS, multiple trajectories, and genes that mark transitions.

## MATERIALS AND METHODS

### Method details

#### Overview of QuanTC

**Figure 1.**
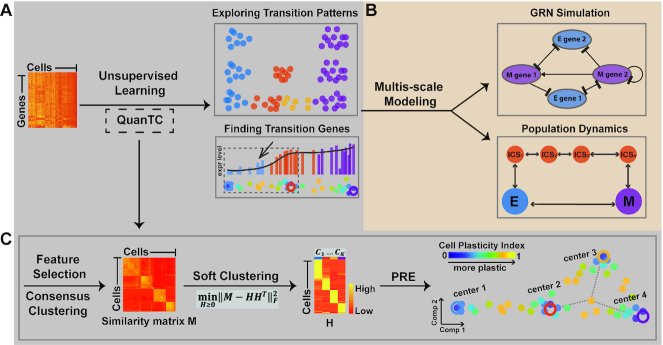
Outline of key components of the approach in analyzing transition cells and ICS. (**A**) Input single-cell transcriptomic datasets to an unsupervised learning method (QuanTC) to explore the transition cells, transition genes and other transition properties. (**B**) Develop multi-scale agent-based of gene regulatory network and cell-population dynamics models to validate and test outputs from QuanTC. (**C**) Overview of QuanTC: 1) feature selection and consensus clustering, 2) calculation of cell-to-cell similarity matrix, 3) computing cell-to-cluster matrix via NMF, and 4) using probabilistic regularized embedding (PRE) for two-dimensional visualization: Each solid circle represents one cell, colored by the value of Cell Plasticity Index (CPI) that quantifies the transition capability of each cell, and each larger circle represents the center of a stable cell subpopulation.

QuanTC takes the scRNA-seq data matrix as input to construct a cell-cell similarity matrix using a consensus clustering method (Figure 1) (20). Via non-negative matrix factorization (31), a method of soft clustering, QuanTC then calculates the probabilities of a given cell belonging to the identified clusters (Figure 1C). To detect transition cells (TC), the cell-to-cluster probabilities are next used to measure the plasticity of each cell, i.e. the extent to which the cell may change its cluster identity. To better visualize cells in transition, we project cells to a low-dimensional space based on a probabilistic regularized embedding (PRE) (Figure 1C). The transition trajectories are then inferred by summing the cluster-to-cluster transition probabilities that are calculated from cell-to-cluster probabilities and TC between clusters. The clusters in the middle of the transition trajectories are denoted as ICS. The transition genes and marker genes of clusters are obtained through factorizing the gene expression matrix as product of cell-to-cluster probabilities and likelihoods of genes uniquely marking each cluster.

#### Feature selection and consensus matrix construction

We start by removing the low-expressed cells (expressed $ < 5\%$ of the total number of genes), and the rare and ubiquitous genes that are either expressed in less than $10\%$ of cells or expressed with low variance ($ < 0.005$) among all cells (Figure 1C). Then we fit expressions of each gene with a Gaussian mixture model consisting of three distributions and use the weights and means of the model to choose the most informative (bimodal distributed) genes. We remove the rarely expressed genes for which the components of the mixture models with mean $0$ accounting for more than $90\%$ weights. To select the bimodal distributed genes, we rank the remaining genes according to two criteria. We first sort the difference between means of the top two components in descending order. Then we sort the difference between weights of the top two components in ascending order. By aggregating the ranks of the two orders, we select the top $3000$ informative genes for further analysis.

#### Quantifying transition cells via cell plasticity index (CPI)

QuanTC computes a cell-to-cell similarity matrix, $M$, through the cluster-based similarity partitioning algorithm to estimate the similarity between cells. A binary matrix is constructed for each clustering outcome such that two cells are classified within one cluster, the corresponding value in the binary matrix is one, otherwise zero. A cell-to-cell similarity matrix $M$ is calculated as the mean of the binary matrices constructed from clustering, leading to a symmetric non-negative matrix.

Through symmetric non-negative matrix factorization (31–34), the cell-cell similarity matrix $M$ is decomposed into a product of a non-negative low-rank matrix $H$ and its transpose ($n$ is the number of cells, $k$ is the number of clusters) (Figure 1C):

(1) $$\begin{equation*}\mathop {\min }\limits_{H \ge 0} \|M - H{H^T}\|_F^2,H \in {R^{n \times k}}.\end{equation*}$$

Each column of $H$ represents a cluster and each row of $H$ corresponds to the relative weights of a cell belonging to all the clusters. In other words, $H$ contains the clustering information of cells: the largest element in each row showing the cluster identity of the corresponding cell and the likelihood of a cell belonging to each cluster. The number of clusters $k$ is estimated by analyzing the largest gap of the sorted eigenvalues of symmetric normalized graph Laplacian (Supplementary Figure S1A).

By normalizing each row of $H$, we obtain a probability-like matrix ${{P\ }} = \ [ {{p_{ij}}} ]$ where ${p_{ij}}$ represents the probability of cell $i$ belonging to cluster ${\rm{j}}$. QuanTC uses an entropy approach to characterize the degree of plasticity of each cell through a Cell Plasticity Index (CPI) (for cell *i*) defined as (Figure 1C):

(2) $$\begin{equation*}{\rm{CP}}{{\rm{I}}_i} = \ - \frac{1}{{\log k}}\mathop \sum \limits_{j\ = \ 1}^k {P_{ij}}\log ({P_{ij}}).\end{equation*}$$

A cell undergoing the transition between clusters has higher entropy in contrast to cells located in one well-defined cluster. A higher value of CPI for a cell implies the cell is more plastic, making transition between clusters.

#### Visualization of transition trajectories

In order to faithfully capture both transition trajectories and discrete cell states, the cells are visualized through a probabilistic regularized embedding (PRE) approach using a probability-like matrix ${{P}}$ in a low-dimensional space (Figure 1C). We first calculate the cluster-cluster relationship from ${H^T}H$, where each row of $H$ denotes to what extend the cells belonging to each cluster while each row of ${H^T}$ defines a distribution of weights over all cells in the cluster. The locations of cluster centers ${a_j}$ in the two-dimensional space are then computed via the projection of the cluster-cluster relationship (35). The projection of cells $x$ is achieved by aligning each cell to the cluster centers based on the probabilities while keeping cells separate from each other through the following constraint:

(3) $$\begin{equation*}\mathop {\min }\limits_X \mathop \sum \limits_{i{\rm{\ }} = {\rm{\ }}1}^n \mathop \sum \limits_{j{\rm{\ }} = {\rm{\ }}1}^k {p_{ij}}\|{x_i} - {a_j}\|_2^2 - \frac{{{\lambda _1}}}{n}\mathop \sum \limits_{i{\rm{\ }} = {\rm{\ }}1}^n \mathop \sum \limits_{l{\rm{\ }} = {\rm{\ }}1}^n \|{x_i} - {x_l}\|_2^2.\end{equation*}$$

The cluster with possible transitions to all the other clusters, which shows strong potential of high plasticity, is considered as a candidate for an ICS. The potential transition trajectory among clusters are then inferred via selecting one of the non-ICS (e.g. epithelial cells) as the initial cluster and ordering the clusters according to transitions. Two clusters are considered as neighbor if there are TC between them. By aligning cells along the potential cluster transition via the probability matrix $P$, QuanTC detects the transition trajectories. A cell $i$ is aligned between cluster $k$ and $j$ if the two largest elements of $i$th row of the probability matrix $P$ are located at $k$th and $j$th columns. The cells aligned from cluster $k$ to $j$ are then ordered in ascending CPI with the largest element at $k$th location and in descending CPI with the largest element at $j$th location. The starting cell is selected as the cell with the largest probability belonging to the chosen initial cluster. In the method, multiple transition trajectories might exist, and the probabilities of occurrence of different transition trajectories are calculated by the percentage of cells included in each trajectory over the entire cell population size.

Furthermore, QuanTC calculates its own pseudotime of cells in each transition trajectory. A cell's pseudotime value is calculated as the Euclidean distance in PRE from the starting cell. In order to make the pseudotime value comparable for cells from different trajectories, we scale the range of pseudotime values between neighboring clusters to obtain a global pseudotime value of each cell by using the minimum value along all possible transition trajectories.

#### Finding cluster marker genes and the transition genes that mark transition

In order to identify the marker genes of clusters, we calculate the probabilities for each gene to uniquely mark a cluster. This is achieved by minimizing the difference between the submatrix ${D_s}$, containing cells from one inferred transition trajectory of the original feature selected gene expression ${D,}$and the submatrix ${H_s}$, with such cells of the factorized matrix $H$ ($m$ is the number of genes):

(4) $$\begin{eqnarray*} \mathop {\min }\limits_{\bar{H},W} \|{D_s} - \bar{H}W\|_F^2 - {\lambda _2}{\rm{Tr}}\left( {{{\bar{H}}^T}{H_s}} \right),\bar{H} \in R_ + ^{n \times k},W \in R_ + ^{k \times m}.\nonumber\\ \end{eqnarray*}$$

The optimization solution leads to a gene-cluster matrix $W$ to ensure that the factor matrix $\bar{H}$ is similar to ${H_s}$ derived from the consensus similarity matrix. Then the gene-cluster matrix $W$ can be used to infer transition genes and marker genes. Each column of $W$, after normalization, describes likelihoods for the corresponding gene to uniquely mark the clusters. Each row of $W$, describes how well the genes delineate the corresponding cluster. The marker genes of cluster $j$ are the genes with the largest values located at $j$th row of the column-normalized $W$. The marker genes of a specific cluster are then ordered based on their corresponding elements in row $j$ of the column-normalized $W$. The difference of the top two elements of each gene is chosen to be greater than a given value (default value is 0.03) to ensure that the gene is differentially expressed in cluster $j$. The default value of ${\lambda _2}$ is 10, and how $W$ depends on the parameter is investigated, showing robustness of the method (Supplementary Figure S1B).

In order to uncover genes that mark the transition, that is, the genes varying most among the transition (Figure 1A), we select the marker genes of the two clusters involved in the transition and calculate the Spearman's rank correlation coefficient between gene expression and the order of cells by CPI undergoing transition. Genes with absolute value of Spearman's rank correlation coefficient above a specified threshold (default value is 0.64) are considered as transition genes for the transition of the two clusters. A positive coefficient implies the gene expression levels of aligned cells show increasing changes while the negative coefficient implies decreasing in gene expressions during transition.

### Multiscale agent-based single-cell model based on gene regulatory network

A multiscale model is constructed to track the gene expression values in each cell using an EMT regulatory circuit of genes (7) that are stochastic in time. 18 ordinary differential equations are used to describe the expression levels over time based on a previous study (7). With certain parameters, the circuit has four distinct stable steady states. Each cell is located at one of the four steady states or makes transition towards those steady states. The transition between different steady states may be caused by external signals or induced by stochastic influences over time. In the model, we make the following assumption:

1. The initial population is composed of 200 cells: 50 epithelial cells (E), 50 first intermediate cells (I1), 50 second intermediate cells (I2) and 50 mesenchymal cells (M).
2. All cells divide at a normally-distributed rate $\sim \ \mathcal{N}( {700,200} )$ ($\mathcal{N}$ refers to a normal distribution). The time unit in the model is hour and the parameter values of the model are chosen based on a previous study (7). Every time a cell divides, its expression levels of all the EMT factors are used as initial conditions to its daughter cells. The gene expression levels of each cell are compared to the expression levels of different stable steady states in the EMT spectrum to determine the cell's phenotype. The E state is characterized by high Ecad expression, and M state is characterized by high Vim expression. I1 and I2 states are characterized by both relatively high Ecad and Vim expression while I1 corresponds to stronger Ecad expression and I2 corresponds to stronger Vim expression among the stable steady states (Supplementary Figure S2A). The cells not at any steady states are considered as TC.
3. Stochastic effects are integrated into our model by adding two types of noise (Supplementary Figure S2B). (a) we first perturb the expression levels of the mother cell upon its division into two daughter cells:
  $$\begin{equation*}\ nois{e_{div}} = I_{expr}^{mother}\ {\rm{*}}\mathcal{N}\left( {0,0.7} \right)\end{equation*}$$
  $$\begin{equation*}\ I_{expr}^{daughter1} = I_{expr}^{mother}\ + nois{e_{div}}\end{equation*}$$
  $$\begin{equation*}\ I_{expr}^{daughter2} = I_{expr}^{mother}\ - nois{e_{div}}\end{equation*}$$
  In this case, the noise added at the division is the expression levels of mother cell multiplied by a normally-distributed rate. The perturbed expressions serve as the initial conditions for the daughter cells. (b) The multiplicative noise is applied to the parameters in the EMT model:
  $$\begin{equation*}d{I_{expr}} = \ f\left( {{I_{expr}}} \right)dt + \sigma {I_{expr}}d{W_t}\end{equation*}$$
  The function $f$ represents the EMT regulatory circuit dynamics and $W$ stands for the Wiener process with $\mathbb{E}{W_t} = \ 0$ and $\mathbb{E}{W_t}{W_s} = \ {\rm{min}}( {t,s} )$. ${\rm{\sigma }}$ represents the noise amplitude with default value 0.01. We use Euler-Maruyama scheme to numerically solve the system.
4. The number of times a cell can divide is described by a discrete uniform distribution $\sim \ \mathcal{U}( {2,7} )$ with an equal probability chosen from a natural number between 2 and 7. Once the cell cannot divide any more, the cell dies at a normally-distributed rate $\sim \ \mathcal{N}( {1000,100} ).$

The multiscale model is simulated over a time span of five cell division cycles.

#### Dynamical system modeling of transition trajectories and three dynamic quantities

To reduce the parameter complexity and increase model accountability, we simplify the model to incorporate only three dimensionless parameters ${\rm{\alpha }},{\rm{\beta }}$ and $\gamma$ (Supplementary Figure S3). For easy comparison, the direct transition rate (DTR) from E to M state is used as a base for comparison (set to one). The parameter ${\rm{\alpha }}$ represents the dimensionless cell-state transition rate from M state directly to the E state (i.e. the reverse DTR). We assume that ${\rm{\alpha }} >1$ to guarantee that E state is more stable at equilibrium when there is no induced EMT by extrinsic signal. It also incorporates the effects of other possible M-to-E transitions (MET) that might not be revealed by the trajectories in EMT datasets. The parameter $\gamma$ depicts the forward transition rate between adjacent cell states along the ICS-regulated transition path, also denoted as the indirect transition rate (IDR) of EMT. We use$\ {\rm{\beta }}\gamma$ to represent the reverse cell-state transition rates along the indirect EMT routes with ICS. Based on the inferred transition paths (Results), we assume that $\gamma \gg 1$ and ${\rm{\beta }} \ll 1$ such that EMT is mainly carried out through the ICS-regulated trajectories, and the rate of EMT is significantly larger than the reverse MET along these trajectories.

Then the prescribed ordinary differential equations (ODEs) that describe the population fraction change of epithelial $E( t )$, mesenchymal $M( t )$ and ICS ${I_k}( t )( {k\ = \ 1,2,\ldots,N} )$ can be derived.

(5) $$\begin{equation*}\frac{{{\rm{dE}}}}{{{\rm{dt}}}} = {\rm{\ \alpha M}} + {\rm{\beta \gamma }}{{\rm{I}}_1} - \left( {1 + {\rm{\gamma }}} \right){\rm{E}},\end{equation*}$$

(6) $$\begin{equation*}\frac{{{\rm{d}}{{\rm{I}}_1}}}{{{\rm{dt}}}} = {\rm{\ \gamma E}} + {\rm{\beta \gamma }}{{\rm{I}}_2} - {\rm{\gamma }}\left( {1 + {\rm{\beta }}} \right){{\rm{I}}_1},\end{equation*}$$

(7) $$\begin{eqnarray*} \frac{{{\rm{d}}{{\rm{I}}_{\rm{k}}}}}{{{\rm{dt}}}} = {\rm{\ \gamma }}{{\rm{I}}_{{\rm{k}} - 1}} + {\rm{\beta \gamma }}{{\rm{I}}_{{\rm{k}} + 1}} - {\rm{\gamma }}\left( {1 + {\rm{\beta }}} \right){{\rm{I}}_{\rm{k}}},{\rm{\ }}2 \le {\rm{k}} \le {\rm{N}} - 1,\nonumber\\ \end{eqnarray*}$$

(8) $$\begin{equation*}\frac{{{\rm{d}}{{\rm{I}}_{\rm{N}}}}}{{{\rm{dt}}}} = {\rm{\ \gamma }}{{\rm{I}}_{{\rm{N}} - 1}} + {\rm{\beta \gamma M}} - {\rm{\gamma }}\left( {1 + {\rm{\beta }}} \right){{\rm{I}}_{{\rm{N}},}}\end{equation*}$$

(9) $$\begin{equation*}\frac{{{\rm{dM}}}}{{{\rm{dt}}}} = {\rm{\ E}} + {\rm{\gamma }}{{\rm{I}}_{\rm{N}}} - \left( {{\rm{\alpha }} + {\rm{\beta \gamma }}} \right){\rm{M}},\end{equation*}$$

The initial conditions of ODEs are set as $E\ ( 0 ) = \ 1,M\ ( 0 ) = {I_k} \ ( 0 ) = \ 0$ to assume only E cells initially. To tackle the stiffness problem introduced by large N or ${\rm{\gamma }}$, we called ODE15s solver in Matlab to evolve the dynamical systems.

To study noise attenuation, we add the persistent white noise term to epithelial dynamics, Equation (5) to simulate the extrinsic fluctuation, i.e. we modify the dynamics as stochastic differential equation (SDE)

(10) $$\begin{eqnarray*} {\rm{d\tilde{E}}}\ ( {\rm{t}}) = [ {{\rm{\alpha \tilde{M}}}( {\rm{t}} ) + {\rm{\beta \gamma }}{{{\rm{\tilde{I}}}}_1}( {\rm{t}}) - ( {1 + {\rm{\gamma }}}){\rm{\tilde{E}}}( {\rm{t}})}]{\rm{\ dt}} + {\rm{\sigma d}}{{\rm{W}}_{\rm{t}}},\nonumber\\ \end{eqnarray*}$$

where ${{\rm{W}}_{\rm{t}}}$ is the standard Wiener process with $\mathbb{E}{{\rm{W}}_{\rm{t}}} = {\rm{\ }}0$ and $\mathbb{E}{{\rm{W}}_{\rm{t}}}{{\rm{W}}_{\rm{s}}} = {\rm{\ min}}( {{\rm{t}},{\rm{s}}} )$ and ${\rm{\sigma }}$ represents the noise amplitude, which is set as 1 in our simulation. We use Euler-Maruyama scheme to simulate system described by Equations (6–10).

The mesenchymal population fraction $M( t )$ potentially measures how the EMT process adapts or responds to extrinsic signals or fluctuations, as well as the efficiency of transition from epithelial to mesenchymal cells. To quantify the three properties, in a model with $N$ intermediate states we define adaptation sensitivity (AS), noise attenuation (NA) and transition efficiency (TE) as

$$\begin{eqnarray*}A{S_N} &=& \frac{{\mathop {\max }\limits_t M(t) - M({+ \infty })}}{{\mathop {\max }\limits_t M(t)}}, \nonumber\\ N{A_N} &=& \frac{{{\rm{std}}[ {{\rm{\tilde{M}}}(t)}]}}{{{\rm{mean[\tilde{M}}}(t)]}}\ ,T{E_N} = \ M( { + \infty }) \end{eqnarray*}$$

where ${\rm{\tilde{M}}}( t )$ denotes the mesenchymal population in the stochastic ODEs. The reliance of $A{S_N},N{A_N}$ and ${T}{{E_N}}$ on N and $\gamma$ are investigated to study different EMT lineage structures and role of ICS in population-survival. We explore the AS, NA and TE as the functions of key parameters N and ${\rm{\gamma }}$ (Supplementary Figure S3B-D). From the single-cell data analysis, the embryonic EMT is associated with an increase of ${\rm{\gamma }}$, while in cancer EMT there is a simultaneous increase of N and ${\rm{\gamma }}$.

#### Roles of ICS in adaptation

When the ICS does not exist in the system, the dynamics of M population can be solved explicitly as ${\rm{M\ }}( {\rm{t}} ) = \frac{1}{{1 + \alpha }}\ ( {1 - {e^{ - ( {1 + \alpha } )t}}} )$, which is a monotonic function of time. Therefore, the adaptation sensitivity is zero in the two-state system. Generally, in the linear system Equations (5–9) with N ICS, the solution can be expressed as ${\rm{M}}( {\rm{t}} ){\rm{\ }} = {C_0}\ + \mathop \sum \limits_{k\ = \ 1}^{N + 1} {C_k}{e^{{\lambda _k}t}},Re( {{\lambda _k}} ) < 0$. When the eigen-values$\ {\lambda _k}$ are real and${\rm{\ }}{C_k}$ have different signs, there could exist local maximums of ${\rm{M}}( {\rm{t}} )$ trajectory, resulting in the non-zero adaptation sensitivity. Meanwhile, if the eigen-values$\ {\lambda _k}$ are complex, we even can have the oscillatory trajectory of ${\rm{M}}( {\rm{t}} )$ before it reaches stationary state. Through numerical simulation, we validate that the adaptation sensitivity will increase with N when keeping other parameters as constant (Supplementary Figure S3B).

### Quantification and statistical analysis

#### hESCs data

The single-cell qPCR data (36) was performed with 48 selected genes during a sequential EMT-MET from days 0 to 21. We start with 345 cells from day 0 to day 3. Based on the cell-cell similarity matrix resulting from consensus clustering (20), we use the largest gap of consecutive eigenvalues of symmetric normalized graph Laplacian to infer the number of cluster $k\ = \ 3$. The initial cluster chosen to be the start of transition trajectory because of including day 0 (epithelial) cells.

#### SCC data

We apply QuanTC to the SCC dataset (37) including 382 cells. After removing the low-expressed cells (expressed <5% of the total number of genes), 361 cells remain for further analysis. After feature selection, we use top 3000 genes for consensus clustering and inference of marker genes and transition genes. The cluster having the smallest number of TC around (i.e. low transition taking place) is considered as the start or the end of the transition trajectory. The initial cluster is named as E state based on the high expression levels of Epcam. Other clusters are named based on the inferred transition trajectories compared with the E-I1-I2-M spectrum in EMT. The cell-cycle phase of each cell is determined based on the computed cell cycle scores provided in Seurat (38,39).

#### Mouse embryonic development data

This scRNA-Seq data (40) includes cells from skin (155 cells), lung (176 cells), liver (123 cells), and intestine (173 cells) during E9.5 to E11.5. After removing the low-expressed cells (expressed <5% of the total number of genes), 155 skin cells, 176 lung cells, 123 liver cells and 173 intestine cells remain for future analysis as in SCC data.

#### HNSCC data

This dataset (41) has ∼6000 single cells from 18 head and neck squamous cell carcinoma (HNSCC) patients. We focus on six tumors from which the largest numbers of malignant cell transcriptomes and cells involved in EMT were acquired. The six tumors include patient 5 (132 tumor cells), patient 6 (123 tumor cells), patient 17 (330 tumor cells), patient 18 (140 tumor cells), patient 25 (209 tumor cells) and patient 28 (138 tumor cells). For each patient, we first use all the tumor cells, based on the selected features by QuanTC, for clustering. Similar to the original study (41), we remove the clusters having high expression levels of the cell cycle and stress markers because those cells are known not involved in EMT. For the remaining tumor cells, mostly similar to epithelial cells, we add 20 fibroblast cells to each dataset to act as a reference of mesenchymal cells. We then apply QuanTC to the mixed datasets of each patient. We notice that all the six datasets have four clusters including two ICS. The raw and filtered datasets are available on the package website (https://github.com/yutongo/QuanTC).

#### Mouse hematopoietic progenitors data

This scRNA-Seq data (42) includes 2018 cells. After removing the low-expressed cells (expressed <5% of the total number of genes), 1957 cells remain for further analysis. Twelve clusters are identified by QuanTC (Supplementary Figure S4A). The cells with high CPI values (>0.34) are considered as TC (Supplementary Figure S4B). Cluster C6, C7 and C12 are considered as non-ICS or a potential start or end of the transition trajectories because fewer TC exist in or around them (Supplementary Figure S4C) with weak capability of making transition. B cells and plasmacytoid dendritic cells (pDC) share a common progenitor (42). Cluster C6, C7 are B cells and pDC, respectively, based on the high expressions of the known marker genes (*Ebf1*, *Irf8* and *Siglech*). Based on the relative number of TC between clusters (Supplementary Figure S4D), the transition trajectories C5–C8–C7 and C5–C11–C6 indicate that B cells (C6) and pDC (C7) share a common progenitor C5. The transition trajectories inferred by QuanTC are consistent with the previous findings (42). QuanTC identifies the maker genes and transition genes involved in the two transition trajectories (Supplementary Figure S4E). When ordering cells in the transition trajectories, the known lineage markers increase along the pseudotime (Supplementary Figure S4F).

#### Gene Ontology enrichment

The Gene Ontology enrichment analysis (43–45) is performed on the top 100 markers genes (Supplementary Table S2) of each ICS selected by QuanTC.

#### Comparison with Monocle 3

Monocle 3 (46) is applied to the simulation and SCC datasets (Supplementary Figure S5). While Monocle 3 separates Epcam^+^ tumor cells from Epcam^−^ tumor cells in SCC dataset, it is unable to obtain the known epithelial to mesenchymal lineage (Supplementary Figure S5A). However, if only using the top 3000 genes selected by QuanTC (Supplementary Figure S5B), Monocle 3 is able to capture the previously observed epithelial to mesenchymal lineage, suggesting usefulness of QuanTC in feature selection. For the simulation dataset, Monocle 3 separates different cell states, however, it cannot identify TC, consequently cannot obtain the transitions between clusters (Supplementary Figure S5C).

## RESULTS

Our study consists of two major components: a) unsupervised learning of scRNA-seq data and b) modeling the inferred EMT dynamics (Figure 1). To scrutinize the transition of cells, we first propose QuanTC (Figure 1C, Materials and Methods), a method to quantify the transitional status of individual cells and identify the *transition genes* that mark the transition process and the *marker genes* that distinguish different cell states. The QuanTC is then validated on a multiscale agent-based stochastic model based on a core EMT gene regulatory network (Figure 1B). By applying QuanTC to twelve published single-cell datasets during embryogenesis or cancer, we reveal the common cell lineage structures mediated by the ICS. We finally model such cell lineages (Figure 1B) to investigate similarity and difference of identified cell lineages in terms of signal adaptation, noise attenuation and EMT transition.

### QuanTC faithfully captures cell plasticity and transition trajectory in simulated datasets

**Figure 2.**
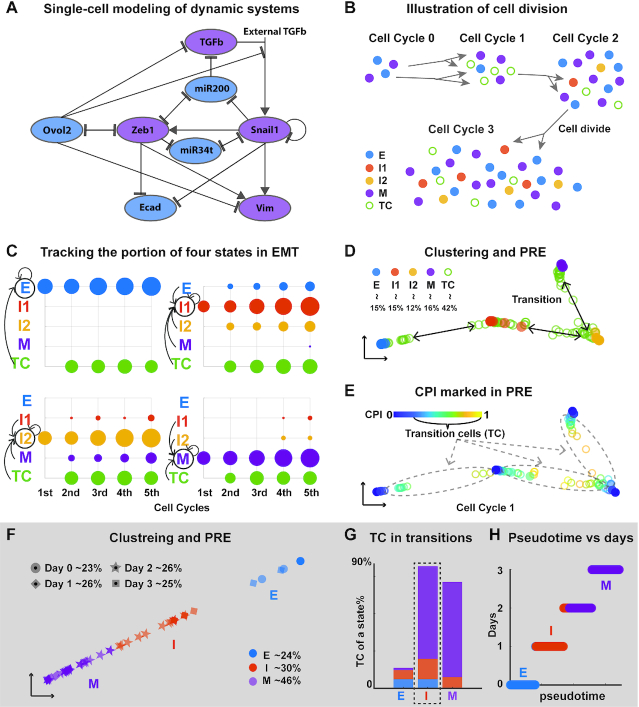
Testing QuanTC on simulated EMT datasets and a qPCR dataset for hepatic differentiation of hESCs. (**A**) The EMT gene regulatory network used in the multi-scale agent-based model; blue: epithelial promoting factor; purple: mesenchymal promoting factor. (**B**) Illustration of the modeling output: each cell colored by its true state labels. (**C**) A simulation dataset: the proportion of each state induced by the previous cell states at the end of each cell cycle. The size of the dot is proportional to the number of cells, and the color denotes the cell states of the mother cell. The arrows represent the occurred state transitions and the circle represents the state of the daughter cell. It shows the transition dynamics of each state. (**D, E**) PRE visualization of each cell at the end of first cell cycle (a circle) colored by its true state from the model (D) and the calculated CPI value (E). The percentage for each cell type is the percentage of a given cell type over the entire cell population size. (**F**) Clustering and PRE visualization of the qPCR dataset. Each dot represents one cell colored by the identified state, and its shape represents its real time. (**G**) Percentage of TC in each state relative to the total number of TC with colors consistent with (F*)*. Dashed box: the intermediate cell state. (**H**) Comparison of the inferred pseudotime and the day collected in the experiment of each cell. The parameters are provided in Supplementary Table S1.

To test capability of QuanTC in capturing transition cells and intermediate cell states, we first constructed a multiscale single-cell model using a core EMT/MET gene regulatory network (Figure 2A) (5,7,10,13,47). The new agent-based model dynamically describes the expression levels of genes featured in the regulatory circuit within individual cells, and explicitly includes cell division to track the individual cells. The cell state transition may be caused by the external signal (TGF-$\beta$) or stochastic effects in cell division and/or gene regulatory dynamics (Supplementary Figure S2B). The single-cell model outputs a group of single cells along with the expression values of the 18 modeled regulatory components at each temporal point (Materials and Methods) to mimic an EMT scRNA-seq dataset.

One typical model simulation exhibits four distinct stable steady states corresponding to four cell phenotypes: epithelial state (E), two intermediate cell states (I1 and I2) and mesenchymal state (M) (Figure 2B). The intermediate state closer to the E is denoted as I1, and the one closer to the M as I2. The cells that have not reached any of the steady states are considered as transition cells (TC). In this simulated system, initially each state consists of 50 cells and after five cell cycles the system grows to 2030 cells. To detect possible transitions between the different states, the cells at the end of each cell cycle were tracked back to the previous cell cycle to identify their mother cells (Figure 2C and Supplementary Figure S6A). For example, E cells were found to come from TC whereas M cells came from TC with few from I1 and I2. The observed transitions among the four states indicate that TC have the strongest capability to give rise to all different EMT subpopulations with the cells in ICS next in such transition capability. Interestingly, E and M cells show less potential to make transitions directly (Figure 2C and Supplementary Figure S6A).

The simulation dataset provides the true label of each cell and its transition details. Applying QuanTC to the data collected at the end of the first cell cycle, we identified four cell states and TC between them (Figure 2D, Materials and Methods). Principal component analysis (PCA) was unable to separate different states at the end of later cell cycles let alone detecting the potential transitions between states (Supplementary Figure S6D, E). To quantify the transition capability, we computed cell plasticity index (CPI) of all cells (Figure 2E) and found that the TC marked using modeling data have relatively high CPI values while cells closer to the primary states have lower CPI values. More TC with higher CPI values were found to be around the two ICS (Supplementary Figure S6B, D, E), suggesting high transition potential of ICS.

The transition genes that mark the transition processes between states, and the marker genes of identified states were uncovered using QuanTC (Supplementary Figure S6C). Ecad and ZEB, along with other genes sharing the similar expression behavior, were found to be marker genes of E and M cells. As for ICS cells, while no clear state marker genes were identified, multiple transition genes are highly expressed due to their strong potential to make transitions (Supplementary Figure S6C).

Through cell state identification, estimating cell plasticity, and inferring marker and transition genes, QuanTC recapitulates the observed states and their transitions in the single-cell model that can be explicitly delineated.

### A near synchronous EMT though one ICS during embryonic stem cell differentiation

Previous studies revealed a global epithelial–mesenchymal–epithelial transition during the hepatic differentiation of human embryonic stem cells (hESCs) (48). Recently, a single-cell qPCR analysis with 48 selected genes was performed to study this process (36). In this dataset, cells from day 0 are all epithelial cells in a pluripotent state while cells at day 3 are definitive endoderm (DE) cells in a typical mesenchymal-like status. Cells from day 0 to day 3 are found to follow a near synchronous EMT.

We applied QuanTC to the dataset of 345 cells from day 0 to day 3, identifying three clusters (Figure 2F). Two clusters are E (high expression of pluripotent marker gene *SOX2*) and M (high expression of DE marker genes *FOXA2* and *GATA6*) whereas the other expresses both epithelial marker gene *CDH1* and DE marker gene *FOXA2* (Supplementary Figure S7), named as intermediate state I.

Next we quantified the transition dynamics of EMT in embryonic stem cell differentiation using QuanTC. We found that the cells located around the overlapping space between clusters have higher CPI values, while cells closer to cluster centers have lower CPI value (Supplementary Figure S7A). More TC with higher CPI values locate around the identified state I, suggesting that the I state has high potential to make transitions to both E and M (Figure 2G). The transition trajectory from E to M via I state includes 99.7% of total cells, indicating that the ICS-mediated path dominates the cell transitions during EMT.

The cells in early pseudotime were found to be the same ones in early real time (Figure 2H), suggesting the transition from day 0 to day 3 follows a near synchronous EMT, a result consistent with the experimental observations on differentiation of hESCs to hepatic lineage (36).

Novel transition genes and marker genes of the three states were identified (Supplementary Figure S7B-C). *MIXL1*, the marker of DE, is identified as a transition gene from E-I, because its expression level increases gradually during E–I transition (Supplementary Figure S7D). Two pluripotency markers, *POU5F1* and *NANOG*, and other genes sharing similar expression profiles are transition genes of I–M because of the observed gradual decrease from I to M.

For this dataset, QuanTC not only captures the synchronous EMT but also detects ICS that express both E and M markers. The ICS identified by QuanTC shows strong transition dynamics and ICS-regulated path dominates the cell transitions during EMT.

### Multiple ICS found in mouse skin tumor dataset

To study epithelial-to-mesenchymal transition in cancer (1,49), we applied QuanTC to a skin squamous cell carcinoma (SCC) dataset, in which multiple tumor subpopulations associated with different EMT stages were identified, and some of them displayed hybrid phenotypes that likely represent multiple distinct ICS *in vivo* (37). This dataset of 382 cells on skin tumors contains FACS-isolated epithelial YFP^+^Epcam^+^ tumor cells, which are relatively homogeneous, and mesenchymal-like YFP^+^Epcam^−^ tumor cells, which are more heterogeneous (37).

**Figure 3.**
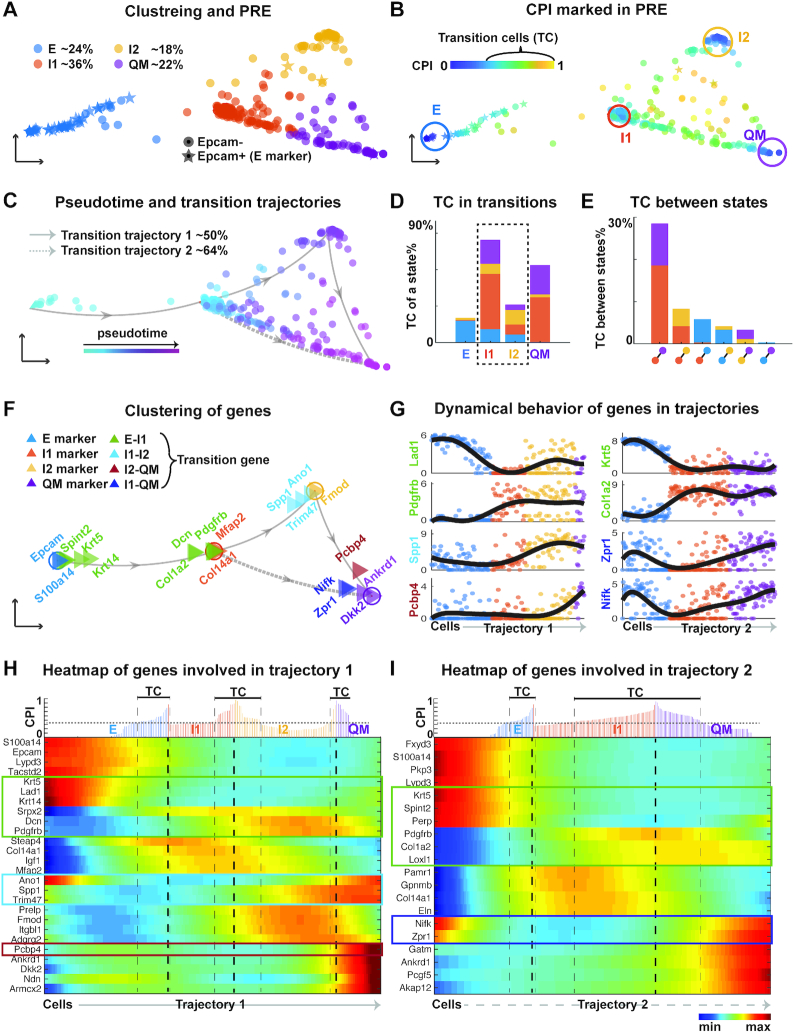
Analyzing EMT in mouse skin squamous cell carcinoma (SCC) dataset using QuanTC. (A–C) Visualization of cells via PRE. (**A**) Each star or solid circle colored by the corresponding cell state represents one of the 67 epithelial YFP+Epcam+ and 292 mesenchymal-like YFP+Epcam- tumor cells. (**B**) Identification of TC. Each dot is colored by its CPI value. The cells outside circles with relatively high CPI values are considered as TC. The parameters are given in Supplementary Table S1. (**C**) Transition trajectory inference. Arrowed solid and dashed lines show two main transition trajectories, with cells colored based on their pseudotime. (**D**) Percentage of TC associated with each state relative to the total number of TC. (**E**) Percentage of TC between two states relative to the total number of cells. (**F**) Visualization of marker genes and transition genes between states. Each triangle represents a gene colored by its type and arrowed lines indicate the transition direction of EMT. (**G**) Expression levels of top transition genes with cells ordered along the two most probable transition trajectories. Solid lines, smoothed expression curves for each gene in the transition trajectory. (**H, I**) Heat map of normalized expression of marker genes and transition genes. Columns represent cells ordered along the transition trajectory and rows represent genes. Coloring represents the normalized expression value of each gene. Transition genes are marked in the box. Top: CPI values of each cell along the transition trajectory.

Four clusters were identified by QuanTC, showing two clusters are clearly E and quasi-mesenchymal (QM) states (Figure 3A and Supplementary Figures S8–S9) and the two other clusters, labeled as I1 and I2, express both epithelial marker gene *Dsp* and mesenchymal marker gene *Vim*. Nearly all epithelial YFP^+^Epcam^+^ cells were found in the E state while most mesenchymal-like cells were clustered into I1, I2 or the QM state. The remaining mesenchymal-like cells were clustered into E but closer to I1, similar to the I1 cells. The overall cell distributions in four different states are very much consistent with the previous observed Epcam^+^ and Epcam^−^ cells in their levels of heterogeneity (37).

Novel transition trajectories from E to QM were revealed according to the locations of TC (Figure 3B). There are two main transition trajectories: E-I1-I2-QM and E-I1-QM, which consist of 94% of cells (Figure 3C). This suggests the two most probable transition trajectories from E to QM both pass through ICS. The I1 and I2 states, consisting of TC from all the other states around them (Figure 3D), show strong capability of making transition—a nature property of cells in intermediate cell state. The transition between I1 and QM was found to have most TC (almost 30% TC in total) followed by the transition between I1 and I2 (Figure 3E).

The identified marker genes of E (Figure 3F-I) have a broad agreement with known markers of epithelial cells (50) (Supplementary Figure S9), with their levels of transition genes varying significantly during transition. For example, *Lad1* decreases gradually and *Pdgfrb* increase gradually as E cells transition to I1.

Using QuanTC we identified new marker genes for ICS, with some of them shown to have special functions in EMT via separating ICS from the mesenchymal-like states. For example, *Igf1* and *Mfap2*, differentially expressed in I1 state, have been shown to induce EMT in hepatocellular carcinoma and in gastric cancer cells respectively (51,52). As a result, ICS can be identified not only via co-expression of epithelial and mesenchymal markers but also through specific ICS markers.

The two ICS, I1 and I2 states, are indeed distinct cell states based on the Gene Ontology enrichment analysis of the top marker genes of I1 and I2 states. Both I1 and I2 states share similar biological processes including cell migration and cell motility (mesenchymal features), in addition to proliferation and cell-to-cell communications (Supplementary Table S2). The ability of regulating cell communication and signaling is uniquely found for ICS. I1 state not only has all the biological processes included in I2 state but also has the unique biological processes related to cell adhesion that shares with the epithelial cells. This suggests that the cells in ICS display hybrid epithelial/mesenchymal features (11) as well as communicates with other cells through cell signaling (9,53).

### EMT via ICS during mouse embryonic development

**Figure 4.**
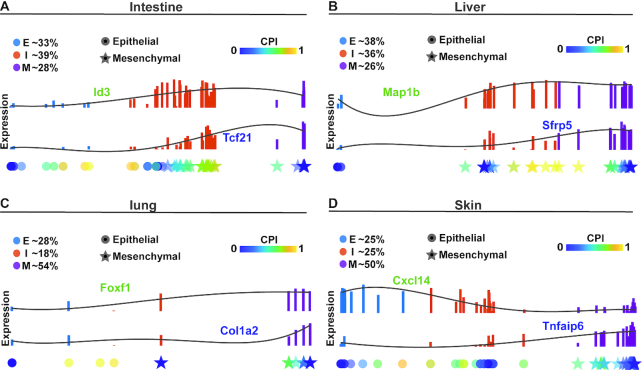
Comparison analysis of EMT during organogenesis in intestine, liver, lung and skin. (**A–D**) Top: the expression levels of E-I transition genes (green) and I-M transition genes (blue) along the E–I–M transition colored by inferred state of cells. Solid lines are smoothed expression curves for each gene in the transition trajectory. Bottom: Cells are ordered along a line according to their pseudotime values. Each dot represents a single cell shaped by the cell states previously identified in the original study on the corresponding dataset and colored by the CPI value. The parameters are given in Supplementary Table S1.

scRNA-seq datasets were collected for four organs and tissues of E9.5 to E11.5 mouse embryos: skin (155 cells), lung (176 cells), liver (123 cells), and intestine (173 cells) (40). Applying QuanTC to the four datasets, three clusters were observed for each dataset (Figure 4). Based on the known cluster labels of epithelial and mesenchymal cells (40) and the marker genes inferred by QuanTC, two clusters are clearly E and M cells (Figure 4 and Supplementary Figures S10–S11). The remaining cluster is located between E and M, with more TC of higher CPI values around it, showing clear characteristics of ICS. The cells close to the I state matches the known labels well, exhibiting mixture of features of epithelial and mesenchymal cells (40).

In the four datasets, >86% cells were found to be involved in the newly discovered E-I-M transition trajectory, suggesting most cells undergoing EMT via the intermediate cell state instead of direct transition from E to M (Supplementary Figures S10–11A, G). Except for skin having only a few more TC in E–I than I–M transition, the other three have significantly more TC in I–M transition than E-I transition (Supplementary Figures S10–11D, J). This observation suggests that I and M states are potentially more similar to each other whereas E could be a distinct state.

Gene Ontology enrichment analysis of the top marker genes (Supplementary Table S2) indicates that the ICS from intestine and liver share several biological processes, including cellular component movement, cell motility and cell migration (mesenchymal features), cell adhesion (epithelial features), regulation of signal transduction and cell communication. The ICS from lung and skin relate to the mesenchymal and epithelial cell differentiation. Interestingly, the transition genes inferred from the four organs or tissues are quite different (Supplementary Figures S10–11), indicating that genes regulating EMT may vary under different conditions at different developmental stages.

### Comparisons with another state transition method and inference of gene regulatory networks

**Figure 5.**
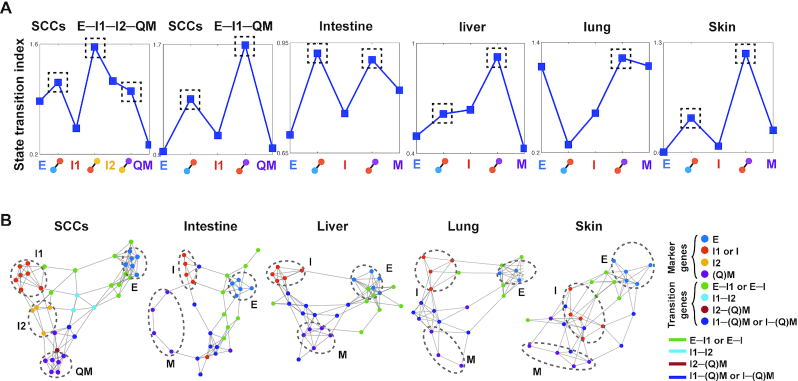
State transition index and gene regulatory networks for five EMT datasets and their comparisons with QuanTC outputs. (**A**) State transition index of relatively stable cells in each state and the TC between states. Dashed box: TC with high value of state transition index. (**B**) Gene regulatory networks of top marker genes and transition genes using the PIDC algorithm from the SCC and mouse embryonic development datasets (the top ∼80% of edges are shown). The parameters are given in Supplementary Table S1. Each dot represents a gene colored by its type. Each large dashed circle labels marker genes of a particular cell state. Graph edges indicate the top interactions and the length of the edge is inversely proportional to the interaction strength between genes.

To further investigate the transition in EMT and validate QuanTC, we next used a previously developed state transition index ${I_c}$ to predict transitions based on a different method that uses correlated information between cells and genes (54). The index ${I_c}$ serves as an early warning signal of a critical transition that coincides with lineage commitment (54). By evaluating ${I_c}$ for all five datasets, we found nearly all TC identified via QuanTC admit higher ${I_c}$ than the cells in the stable states (Figure 5A), consistent with the observation that TC are the cells involved in the transition process. The relatively low cell–cell correlation and high gene-gene correlation (Supplementary Figure S12A) during state transitions correspond to the idea that the state transition involves a decrease of cell–cell correlation and concomitant increase of gene–gene correlation. One exception happens for the E–I trajectory in lung, partly due to a very small number of TC cells (only three cells) identified between E and I.

To investigate how transition genes may regulate state marker genes in EMT, we inferred gene regulatory networks of both state marker genes and transition genes via the PIDC algorithm (55). The inferred markers of different states were projected into lower-dimensional space, with top genes marked by their states or transition trajectories and the edge length, which is inversely proportional to the interaction strength between genes (Figure 5B and Supplementary Figure S12B). Two genes that are close to each other with a short edge indicate a strong regulatory interaction, in contrast to genes located away from each other with a longer edge between them.

For example, in the SCC dataset, E markers are mostly linked to I1 markers through E-I1 transition genes, and marker genes of I1 and I2 are linked directly or via I1-I2 transition genes, showing a gene regulatory circuit consistent with the inferred trajectory and CPI values using QuanTC (Figure 3B, C). In addition, marker genes of I2 and QM are linked directly or via I2-QM transition genes along with an edge linking markers of I1 and QM to I1-QM transition genes nearby, suggesting that E-I1-QM is another transition trajectory, consistent with the two previously inferred trajectories (Figure 3C). Interestingly, markers of E have longer edges linking to other marker genes, suggesting the relative dissimilarity of E to I1, I2 and Q, consistent with our findings directly using QuanTC (Figure 3). Similar structures in gene regulatory networks were seen among the intestine, liver and lung. In particular, marker genes of E, I and M form distinct groups and markers of E and I are linked directly or via E–I transition genes, while markers of I and M are linked directly or via I-M transition genes. Interestingly, for skin, different markers are much less separated compared to other three embryonic development systems, except for markers of E, suggesting the transitions and the genes regulating the transition in developing skin could be more intermingled and complicated.

### Dynamical properties of inferred ICS-regulated EMT trajectories

**Figure 6.**
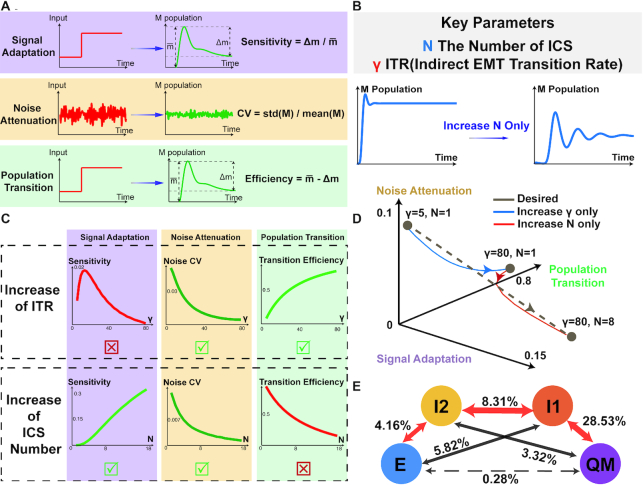
Dynamical properties of inferred ICS-regulated EMT trajectories. (**A**) The definitions and measurements of three quantities – adaptation, noise attenuation and population transition properties of cell population dynamics. (**B**) The key parameters of model including ICS number N and ITR gamma (see also Materials and Methods, Supplementary Figure S3). Increase of ICS number N can result in the multiple peaks in M population trajectory, forming the oscillatory adaptation. (**C**) Effect of tuning N and gamma on the three quantities (see also Supplementary Figure S3). (top row) Changes in three quantities by fixing *N* = 2 and tuning gamma from 5 to 80. The increase in ITR gamma lowers the noise coefficient of variance (CV) of output M population, and increases the transition efficiency from E to M. The signal adaptation sensitivity is not a monotonic function of gamma, which reaches the peak before a certain threshold and declines afterwards with further increase in gamma. (bottom row) Change of three quantities by fixing gamma and tuning *N* from 1 to 18. The increase in *N* improves adaptation sensitivity and noise attenuation, however reducing the value of transition efficiency. (**D**) Tuning parameter gamma and N separately cannot achieve all the desired properties (i.e. simultaneous increase of adaptation sensitivity, noise attenuation and EMT efficiency, indicated by brown dashed line). The desired properties can be achieved by increasing ITR gamma (blue line, increase gamma from 5 to 80 and fix *N* as 1) first and increasing N subsequently (red line, increase *N* from 1 to 8 and fix gamma as 80). (**E**) EMT trajectories inferred from SCC dataset, with node colors consistent with Figure 3. Other inferred trajectories are shown in Supplementary Figures S12–S13. The arrow represents potential transition between states, and number represents the percentage of TC. The red arrows indicate the major transition trajectory mediated by ICS, and the dashed arrow refers to the direct transition route from E to QM state.

To explore the dynamics of the inferred transition trajectories, we developed a cell population model that contains multiple ICS and only relies on three effective dimensionless parameters (Materials and Methods, Supplementary Figure S3A). Subsequently, three emergent quantities were then defined to measure the EMT population dynamics (Figure 6A, Materials and Methods): (i) sensitivity of signal adaptation, (ii) coefficient of variance (CV) to quantify noise attenuation and (iii) the efficiency of population transition from epithelial to mesenchymal states. We then investigated how the existence of ICS, as well as the transitions via ICS, affect the robustness and efficacy of EMT dynamics using these three quantities.

The *signal adaptation* property is demonstrated by the reset of output level after the response to stimulus in cell populations (Figure 6A). In cancer EMT, adaptation with high sensitivity permits the transient peak of the massive release of malignant mesenchymal population, forming the effective metastasis strategy under the immune regulation. In the two-state system with only pure epithelial or mesenchymal states, we rigorously proved that no adaptation is allowed (Materials and Methods). The modeling results suggest that both the increase in ICS number and the moderate increase in *indirect transition rate* (ITR) via the ICS (Supplementary Figure S3B, Materials and Methods) can increase the adaptation sensitivity (Figure 6C), however, further increase in ITR (over a certain threshold) can instead decrease the sensitivity. Interestingly, the increase in ICS number may result in the oscillatory adaptation of cell population dynamics, i.e. the M population goes through multiple peaks before reaching a steady level (Figure 6B). This potentially provides a ‘hide-and-seek’ strategy for metastatic mesenchymal cells battling with immune systems in cancer.

The *noise attenuation* property depicts the system's capability to reduce fluctuations in population dynamics. Both the increase in ICS number and ITR help reduce the CV of M population trajectories (Figure 6C), stabilizing the dynamics in population transition. The property of *population transition* is quantified by the final fraction of M population that originates from pure E population. The increase in ITR results in boosting of population transition efficiency in EMT, while the increase in ICS number reduces such efficiency.

The trade-off between adaptation sensitivity and transition efficiency were observed in EMT (Figure 6C, D). Although larger ICS numbers may increase adaptation sensitivity, it also impairs the effective transition toward M state (Figure 6C). On the other hand, increasing ITR can boost efficiency while the overly-large value results in a decrease in adaptation sensitivity. Hence, an increase in one parameter only, either ICS number or ITR, fails to optimize all the properties simultaneously (Figure 6D). The transition trajectories may need a combined increase in both ICS and ITR to achieve the desired property, as seen in the inferred SCC transition trajectories (Figure 6E).

The derived relationships between three emergent quantities and EMT population parameters shed light on our findings obtained from single-cell EMT data mining. Based on the percentages of TC between state transitions among all the cells involved in EMT, we quantified the EMT trajectories in twelve single-cell datasets by QuanTC (Figure 6E and Supplementary Figures S12C, S13B), which include six additional head and neck squamous cell carcinoma (HNSCC) datasets (Materials and Methods, Supplementary Figure S13). For all the investigated mouse and human datasets from both normal and tumor tissues, we found that the majority of transitions involve ICS while the direct transition between epithelial and mesenchymal states is relatively rare (Supplementary Figures S12C, S13B). This corresponds to the increase in ITR in the model, resulting in the strengthening of noise attenuation property (Figure 6C), as well as enhancement of adaptation sensitivity (provided that increase in ITR does not over-exceed the observed threshold in Figure 6C). Besides, compared to only one ICS involved in EMT in embryo, cancer EMT has more numbers of ICS. Therefore, in cancer EMT the adaptation sensitivity of population dynamics is further enforced by the presence of multiple ICS, with sacrifice of E-to-M transition efficiency. In comparison, in embryogenesis EMT fewer ICS and the large ITR flux can lead to higher E-to-M transition efficiency, however, at the cost of lower sensitivity of population dynamics adaptation.

## DISCUSSION

By unsupervised learning of transition trajectories in twelve EMT single-cell datasets and multiscale mathematical modeling, we have analyzed transition cells and dynamics of EMT that highlights the transition trajectories mediated by ICS. By investigating several emergent dynamic quantities of describing transitions, we have suggested that the inferred transition trajectories not only attenuate the noise, but also enhance the signal adaptation in EMT. Modeling analysis has indicated cancer EMT trajectories strengthen the signal adaptation, whereas trajectories in embryogenesis EMT is in favor of effective population transition toward mesenchymal states.

Compared with direct clustering (20,28) and pseudotime analysis (56–58) for scRNA-seq data, the unsupervised learning algorithm QuanTC can simultaneously detect the intermediate cell states, and construct transition trajectories via quantifying the cell plasticity. An attractive feature of QuanTC is its soft clustering approach to identify cells in mixed states or undergoing transition between states, a ubiquitous property in many cell fate systems. The projection of cells in PRE marked by CPI for transitions offers a parsimonious and meaningful alternative to analyzing a large number of discrete cell states. To compare with other methods, we have applied the popular pseudotime inference method Monocle 3 to the simulation datasets and SCC datasets (Materials and Methods, Supplementary Figure S5). While Monocle 3 correctly depicts the overall progression of epithelial-mesenchymal transition, it lacks the resolution to distinguish transition cells from other stable cells. In addition, the trajectories inferred by Monocle 3 strongly depends on input gene selections. Interestingly, the features selected by QuanTC could improve the consistency of trajectory inference by Monocle 3 in SCC dataset (Supplementary Figure S5), suggesting usefulness and its broader application of the feature selection function in QuanTC.

Unlike other methods that can only infer marker genes for cell subpopulations, such as a recent random coefficient matrix-based regularization method on identifying transition cells (59), QuanTC can uncover key genes that mark the state transitions. The projection of cells in PRE marked by CPI for transition processes offers a parsimonious and meaningful alternative to analyzing a large number of discrete cell types. Besides, QuanTC is adaptive to the downstream analysis of other soft clustering methods and is applicable to systems beyond EMT. For instance, we applied QuanTC to a single-cell RNA-seq dataset of ∼2,000 mouse hematopoietic progenitors (Materials and Methods, Supplementary Figure S4). We found two prominent non-ICS, i.e. plasmacytoid dendritic cells (pDCs) and B cells, exactly corresponding to the target states identified in the original study (42). The transition cells along the trajectory indicates that pDCs and B cells share the same progenitors, consistent with the findings based on the FateID inference (42).

A multiscale agent-based model of EMT gene regulatory network has been developed to generate simulation data with the ground truth, allowing easy validation of our unsupervised learning method QuanTC. Previous models were mainly focused on the regulation mechanisms of EMT by ODEs with feedback control to identify important agents that are responsible for initiating or suppressing EMT (3,5–7). In those models, cell activities or states defined by changes in gene expressions are confined within each individual cell. We have extended the modeling of EMT to a heterogeneous population of cells, while still incorporating gene regulatory networks, offering a convenient framework to explore cell proliferation by monitoring the changes in gene expressions prompted by interactions between various EMT agents, which is important for cancer studies (23,60,61). Our model explicitly incorporates stochastic effects caused by each cell division (62,63) that may affect cell fates. Our model can also easily incorporate different assumptions on proliferative dynamics of each cell state. For example, we have analyzed a case in which the I1 cells are assumed to be non-proliferative (Supplementary Figure S14) to investigate ICS under cell cycle arrest during EMT (64,65).

Interesting trade-offs among signal adaptation, noise attenuation and effective transition have been observed in modeling analysis. Consistent with previous findings (13), the increase in ICS number during EMT attenuates fluctuations; in addition, boosting the transitions via ICS (i.e. ITR) also plays the similar role in noise buffering. The concept of adaptation sensitivity, previously mainly used for signal transductions (16,18), was introduced in this study to quantify the transient, adaptive dynamics in EMT populations. Such transient property were previously reported in breast cancer cell lines (15), and theoretically studied in the context of non-equilibrium statistical physics. Interestingly, the increase of ITR alone cannot improve adaptation persistently, and the robust adaptation in population dynamics requires both large ITR and multiple ICS, a result consistent with the learned single-cell trajectories in SCC. We reason that the transient peaks in highly-adaptive trajectories ensure adequate release of mesenchymal cells, with the short-lasting times impeding immune systems to efficiently capture and respond timely to metastasis. It is very interesting to note that ICS in EMT are associated with poor prognosis of cancer treatment according to clinical studies (23)– our findings between ICS number and adaptation may serve as the potential explanation from cell population dynamics.

In our study, more efficient algorithms to explore cell-cell similarities will likely improve QuanTC significantly in its speed and ability to learn transition trajectories. The agent-based multiscale model can be further improved by adding new interactions between genes and cell-cell communications over time, and the inclusion of other cell types, such as immune cells, may gain further insights into the functional role of ICS. Overall, our integrative approach provides an initial attempt to bridge single-cell data mining and multiscale modeling to investigate transitions and role of intermediate cell states in EMT.

## DATA AVAILABILITY

All the data analyzed in this paper has been previously published and can be accessed from original publications. hESCs (GEO: GSE70741), SCC (GEO: GSE110357), mouse organogenesis (GEO: GSE87038), HNSCC (GEO: GSE103322) and mouse hematopoietic progenitors (GEO: GSE100037) datasets were downloaded from the Gene Expression Omnibus. The code for QuanTC algorithm is available at https://github.com/yutongo/QuanTC, and the simulation code for multi-scale model is available at https://github.com/yutongo/Multiscale-agent-based-model-of-EMT.

## Supplementary Material

gkaa725_Supplemental_Files
