# Supplementary material for: Inference and multiscale model of epithelial-to-mesenchymal transition via single-cell transcriptomic data
Source: Nucleic Acids Res. 2020 Sep 1;48(17):9505–20. doi: 10.1093/nar/gkaa725 (PMC7515733; doi:10.1093/nar/gkaa725)
Supplement: gkaa725_Supplemental_Files [file gkaa725_supplemental_files.zip › manuscript_SA_supp_0719.pdf]

Supplementary Materials

Inference and Multiscale Model of Epithelial-to-Mesenchymal Transition via Single-cell Transcriptomic Data

Yutong Sha[1], Shuxiong Wang[1], Peijie Zhou[1], Qing Nie[1,2,*]

1 Department of Mathematics, The NSF-Simons Center for Multiscale Cell Fate Research, University of California, Irvine, Irvine, CA 92697, USA.
2 Department of Developmental and Cell Biology, University of California, Irvine, Irvine, CA, 92697, USA.

* To whom correspondence should be addressed, Tel: (949) 824-5530, Email: qnie@uci.edu

The supplementary materials include:

Figures S1 to S14
Table S1

which begin next page.

Supplementary Figure

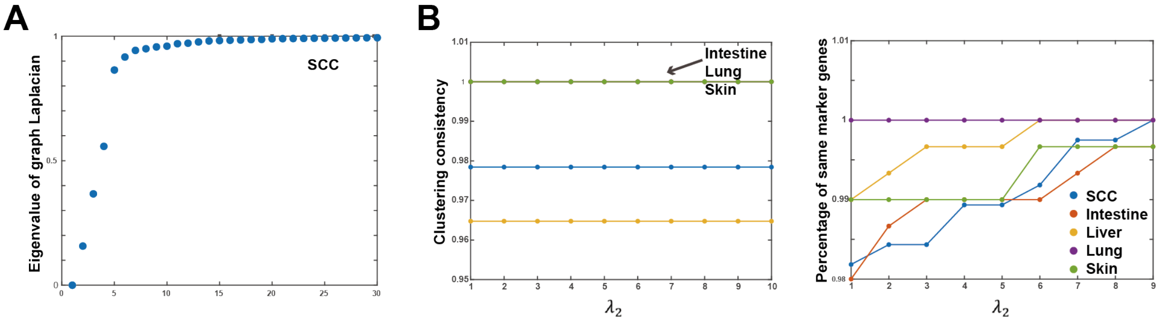

Fig. S1. Number of clusters predicted based on the graph Laplacian and robustness of finding marker genes with varied $\boldsymbol{\lambda_2}$. (*A*) The first 30 sorted eigenvalues of the graph Laplacian of the constructed consensus matrix $\mathbf{M}$ for the SCC dataset. (*B*) Clustering accuracy and robustness of identifying marker genes when $\boldsymbol{\lambda_2}$ varies from 1 to 10 in the SCC and mouse embryonic development datasets. Left: the consistency of clustering results based on the new decomposed $\overline{\boldsymbol{H}}$ compared to the pre-inferred clusters. Right: the percentage of finding the same top 100 marker genes for each cluster compared to the marker genes identified when $\boldsymbol{\lambda_2} = \mathbf{10}$.

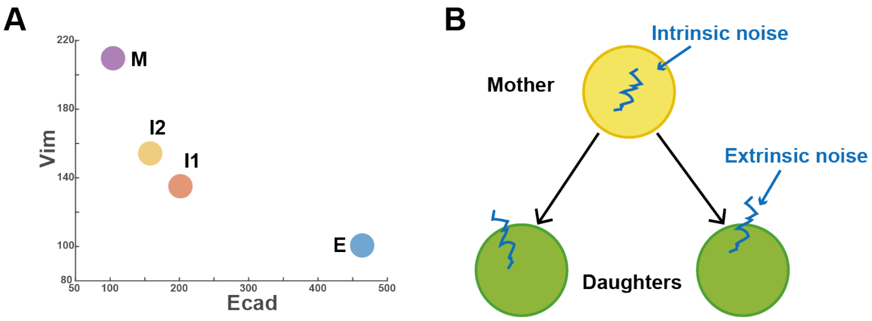

Fig. S2. Modeling illustration. (*A*) Relationship between the four stable steady states and the expression levels of the epithelial marker (Ecad) and mesenchymal marker (Vim) in the model. Each dot represents a stable steady state. (*B*) Illustration of individual cells and cell division: the cell state transition may be caused by the intrinsic noise in gene regulatory dynamics or stochastic effects in cell divisions.

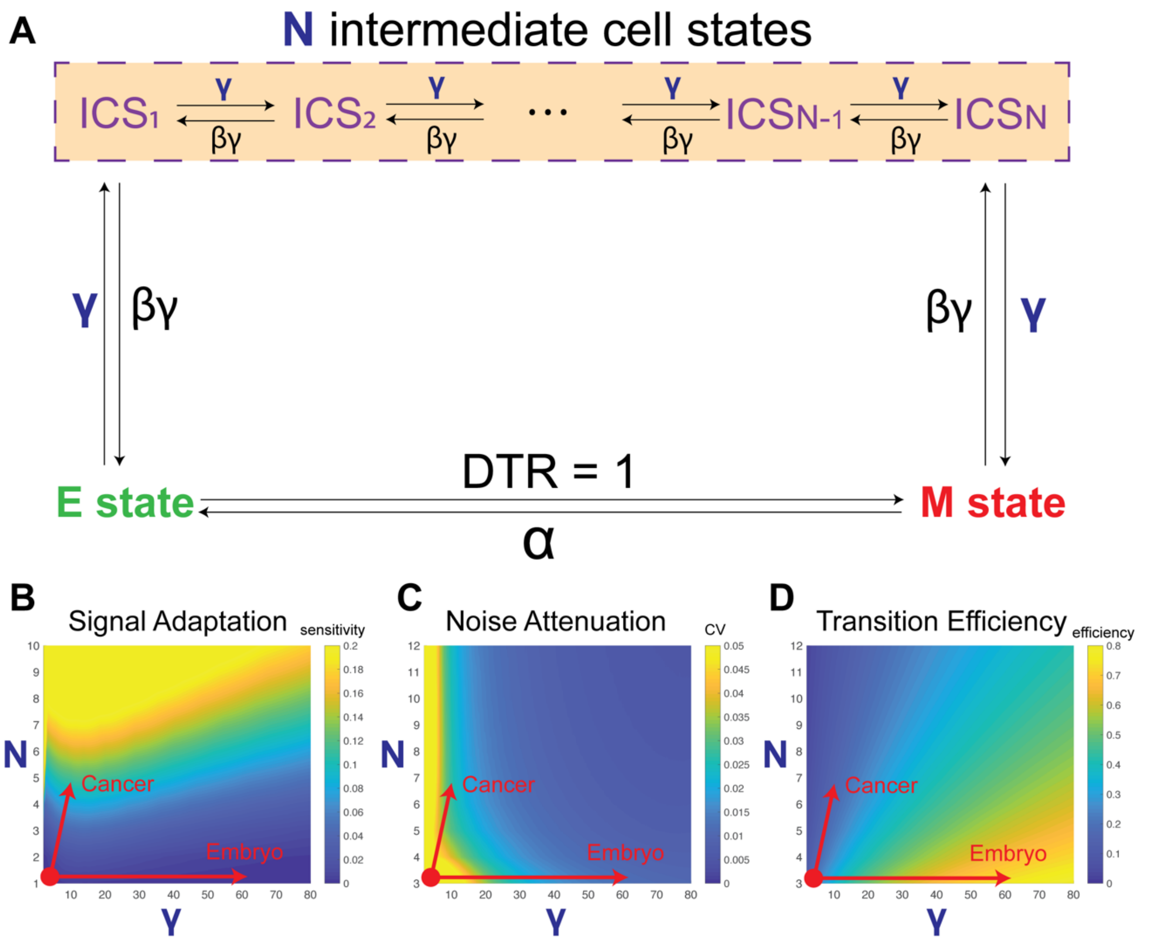

Fig. S3. Mechanism and results of EMT population model. (*A*) The state-transition structure of population model and associated parameters. The model focuses on two major possible routes of EMT 1) the direct transition from E to M state, with rate DTR normalized as 1 and inverse transition rate α. 2) the indirect EMT transition mediated by N ICS, with the forward transition rate (also denoted as the indirect transition rate, ITR) γ and backward transition rate $\beta\gamma$. (*B-D*) The dependence of signal adaptation, noise attenuation and transition efficiency measures over the space of key parameter N and γ. We fix other parameters $\alpha = 10$ and $\beta = 0.01$ in *B-D*. (*B*) The dependence of signal adaptation sensitivity on N and γ. The colors represent the value of sensitivity. The arrows indicate the corresponding transition structures in cancer (increase of both N and γ) and embryo (increase only in γ) respectively. (*C*) The dependence of noise attenutation property on N and γ. The colors represent the CV of output M population dynamics. (*D*) The dependence of transition efficiency on N and γ. The colors represent the value of efficiency.

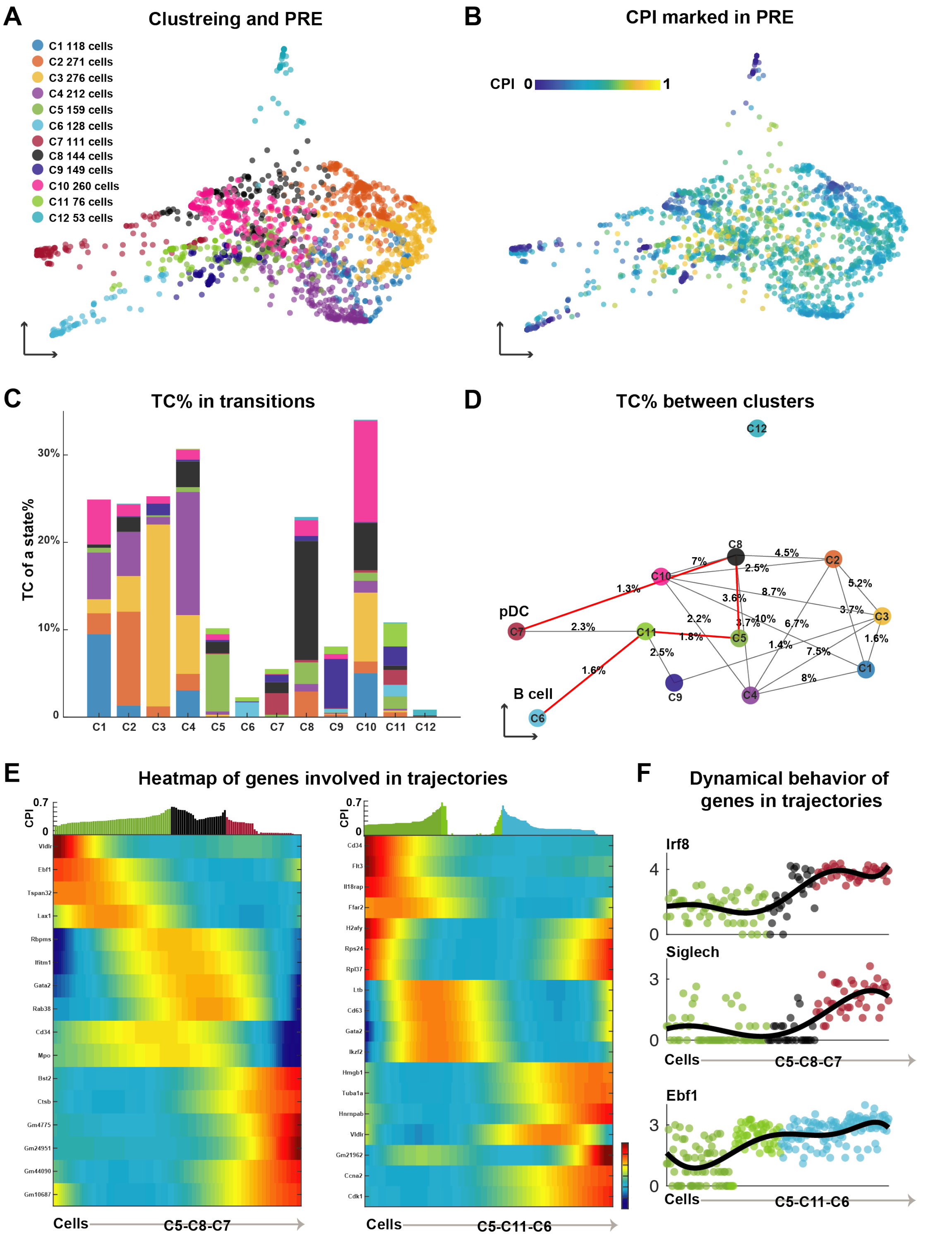

Fig. S4. Analyzing the dataset of mouse hematopoietic progenitors using QuanTC. (*A-B*) Visualization of cells via PRE. (*A*) Clustering result of 1957 cells after preprocess of the data. Each dot represents a cell and is colored by its cluster. (*B*) Each dot is colored by its CPI value. (*C*) Percentage of TC associated with each cluster relative to the total number of TC. (*D*) Percentage of TC between clusters relative to the entire cell population size. The lines show the potential transitions between clusters and the dots located at the cluster centers show the different clusters. Red lines show the potential transition trajectories related to clusters C6

and C7. (*E*) Heatmap of normalized expression of marker genes and transition genes. Columns represent cells ordered along the transition trajectory and rows represent genes. Coloring represents the normalized expression value of each gene. Top: CPI values of each cell along the transition trajectory. (*F*) Expression levels of known lineage markers with cells ordered along the transition trajectories. Solid lines, smoothed expression curves for each gene in the transition trajectory.

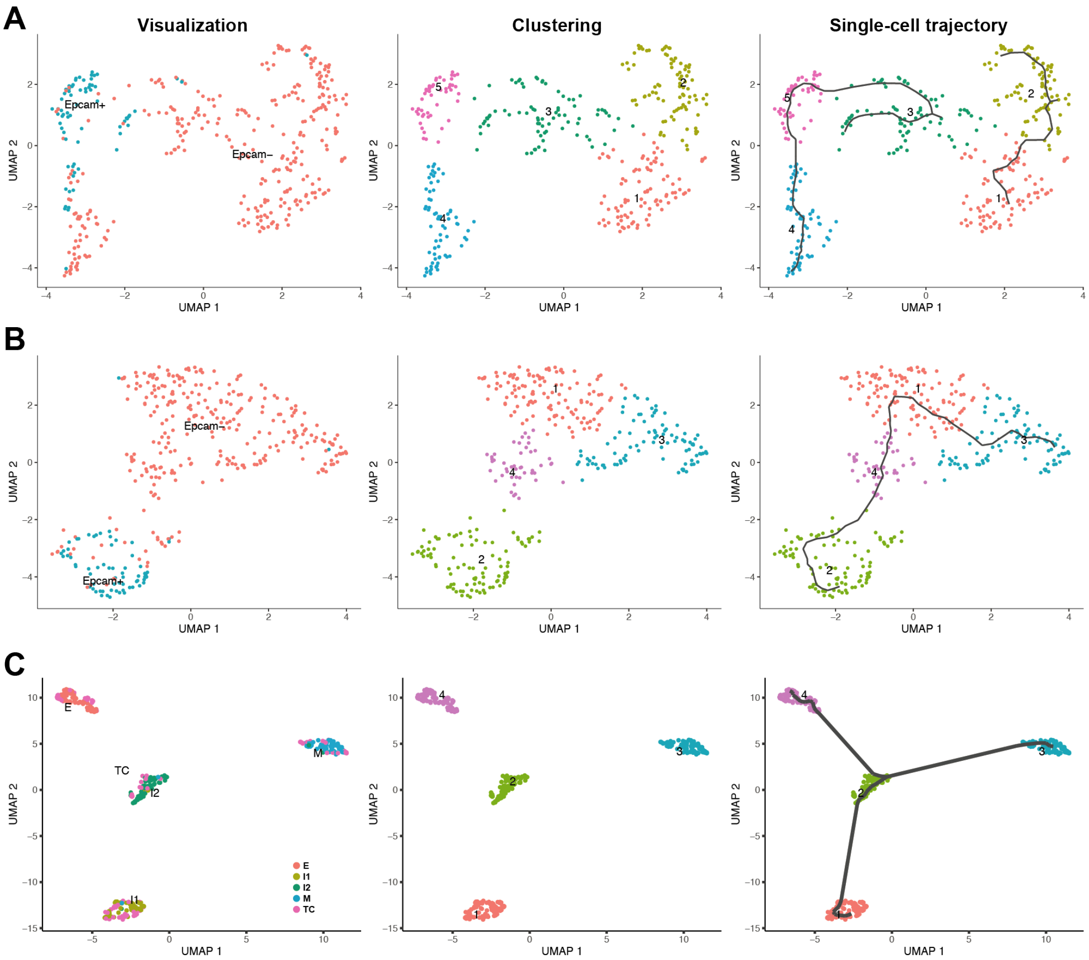

Fig. S5. Analyzing SCC and simulation datasets using Monocle 3. (*A*) Dimensionality reduction of scRNA-seq data using UMAP coloring by the surface marker (left) and by the clusters identified by Monocle 3 (middle, right) using the SCC dataset. Dots represent single cells. Right: Pseudotime-ordering trajectory of scRNA-seq data using Monocle 3. (*B*) Dimensionality reduction of scRNA-seq data using UMAP coloring by the surface marker (left) and by the clusters identified by Monocle 3 (middle, right) using the feature selected (top 3000 genes) SCC dataset from QuanTC. Dots represent single cells. Right: Pseudotime-ordering trajectory of scRNA-seq data using Monocle 3. (*C*) Dimensionality reduction of first cell cycle simulation dataset using UMAP coloring by the known cell types (left) and by the clusters identified by Monocle 3 (middle, right). Dots represent single cells. Right: Pseudotime-ordering trajectory of the data using Monocle 3.

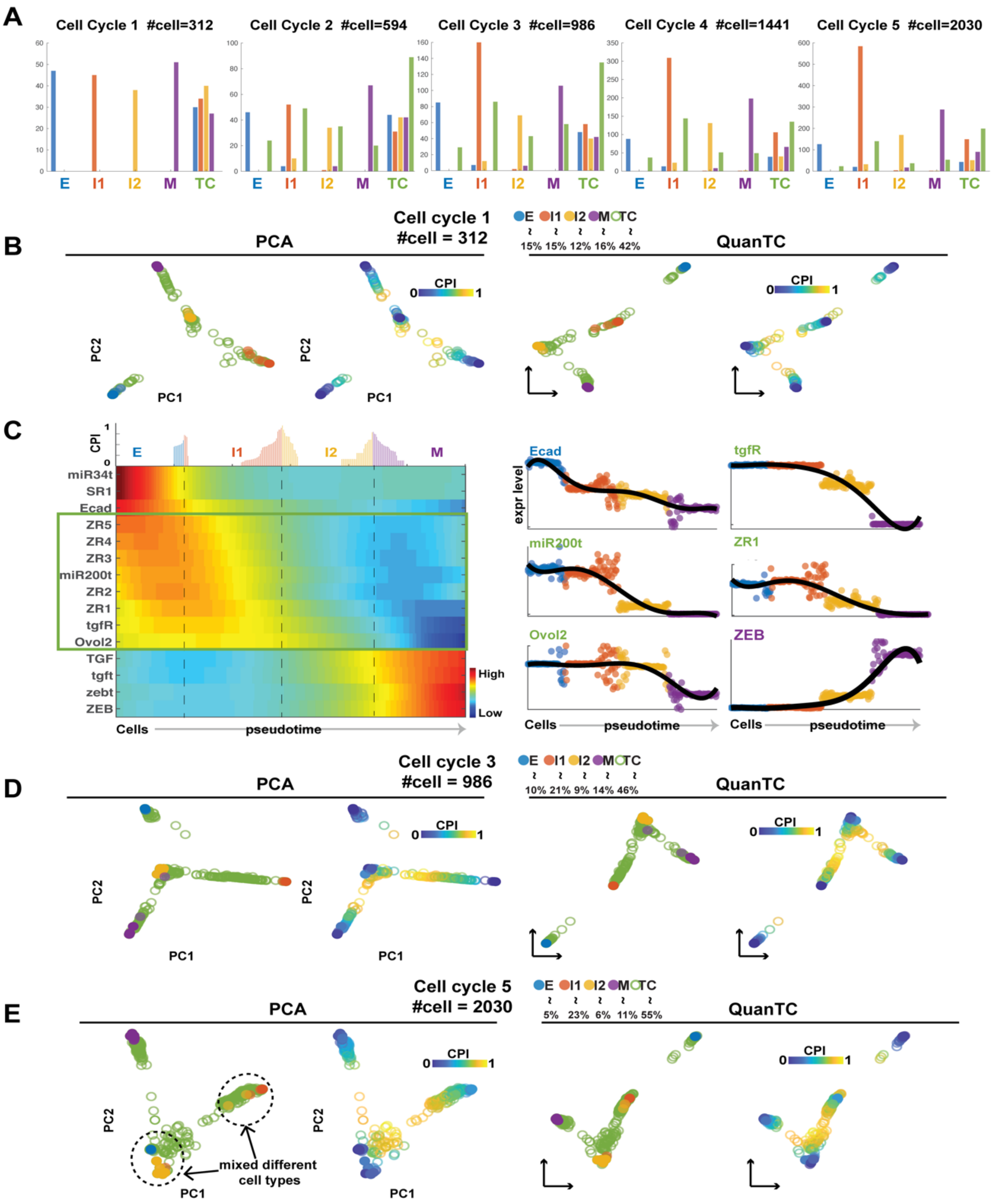

Fig. S6. The distribution of the cell population at the end of cell cycles. (*A*) Histogram of the number of cell population at the end of each cycle. The color denotes the mother cell states. The x-labels represent the states of the daughter cell. (*B-C*) Simulated EMT/MET datasets at the end of first cell cycle. The percentage for each cell type is the percentage of a given cell type over the entire cell population size. (*B*) PCA and PRE visualization of the cells with each cell (a circle) colored by its true state (left) and the calculated CPI value (right). (*C*) Heat map of normalized expression of marker genes and transition genes (left). Columns represent cells ordered along the transition trajectory and rows represent genes. Coloring represents the normalized

expression of each gene. Transition genes are marked in the box. Top: CPI values of each cell along the transition trajectory. Expression levels of top marker genes and transition genes with cells ordered along the most probable transition trajectories (right). Solid lines, smoothed expression curves for each gene in the transition trajectory. (*D-E*) PCA and PRE visualization of the cells with each cell (a circle) colored by its true state (left) and the calculated CPI value (right) from simulated EMT/MET datasets at the end of third (*D*) and fifth cell cycle (*E*).

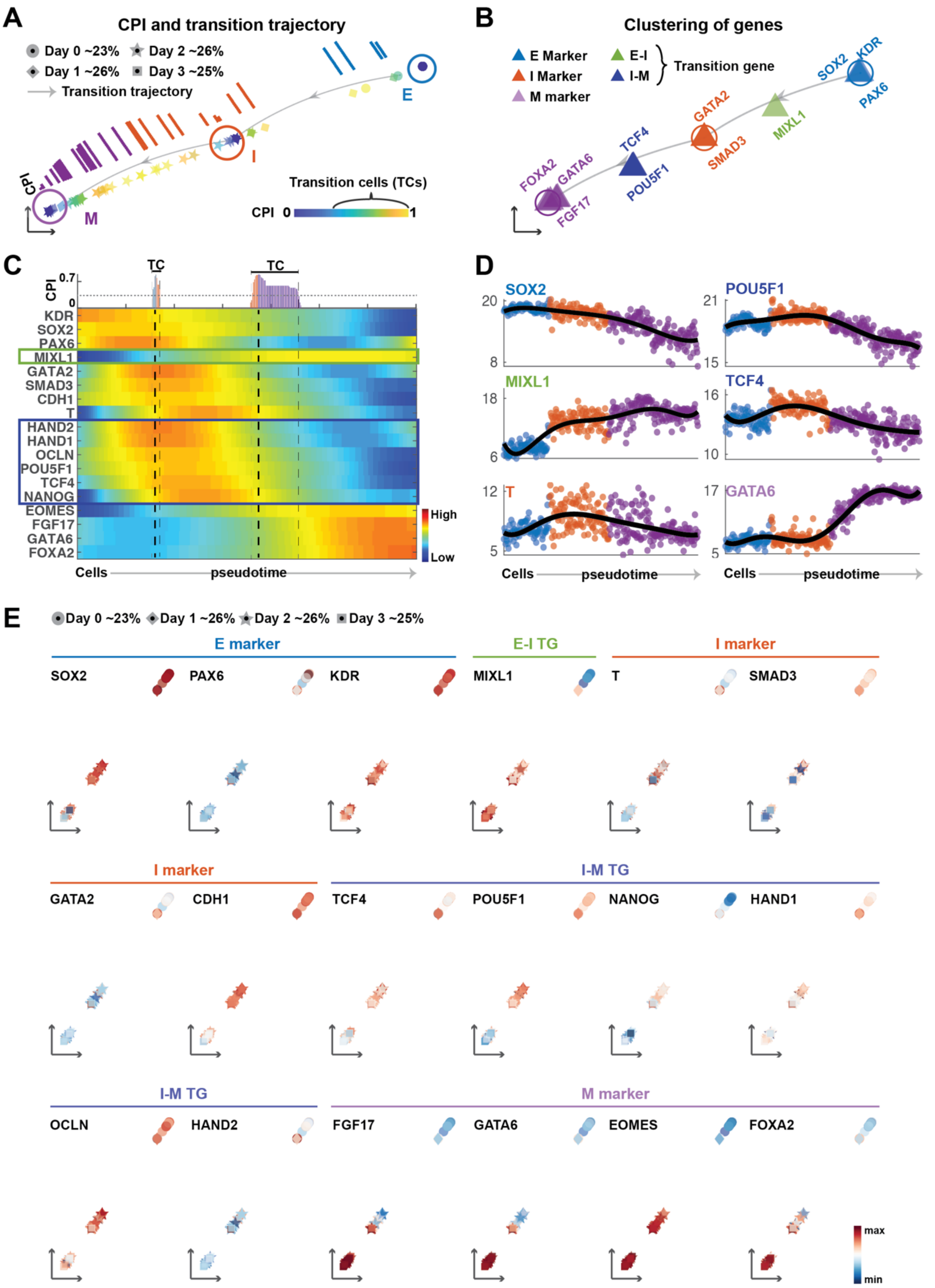

A
CPI and transition trajectory
Day 0 ~23%
Day 2 ~26%
Day 1 ~26%
Day 3 ~25%
Transition trajectory
E
I
M
CPI
Transition cells (TCs)
CPI 0
1
B
Clustering of genes
E Marker
I Marker
M marker
E-I
I-M
Transition gene
SOX2
KDR
PAX6
MIXL1
GATA2
SMAD3
TCF4
POU5F1
FOXA2
GATA6
FGF17
C
TC
TC
CPI
0.7
0
KDR
SOX2
PAX6
MIXL1
GATA2
SMAD3
CDH1
T
HAND2
HAND1
OCLN
POU5F1
TCF4
NANOG
EOMES
FGF17
GATA6
FOXA2
High
Low
Cells
pseudotime
D
SOX2
POU5F1
MIXL1
TCF4
T
GATA6
Cells
pseudotime
E
Day 0 ~23%
Day 1 ~26%
Day 2 ~26%
Day 3 ~25%
E marker
E-I TG
I marker
SOX2
PAX6
KDR
MIXL1
T
SMAD3
I marker
I-M TG
GATA2
CDH1
TCF4
POU5F1
NANOG
HAND1
I-M TG
M marker
OCLN
HAND2
FGF17
GATA6
EOMES
FOXA2
max
min

Fig. S7. Analyzing EMT during hepatic differentiation of hESCs using QuanTC. (*A*) Transition trajectory inference. Each dot represents one cell colored by the value of CPI and its shape represents the time when the data collected given in the original study. The percentage for each cell type is the percentage of a given cell type over the entire cell population size. The cells surrounded by larger circles with relatively low CPI are considered as stable cells. The remaining cells with higher CPI are considered as TC. Arrows indicate the transition direction of EMT. Top, CPI of the cells colored by identified states. (*B*) Visualization of top marker genes and transition genes between states. Each triangle represents a gene colored by its type. Arrows indicate the transition direction of EMT. (*C*) Heat map of normalized expression of marker genes and transition genes. Columns represent cells ordered along the transition trajectory and rows represent genes. Coloring represents the normalized expression of each gene. Transition genes are marked in the box. Top: CPI values of each cell along the transition trajectory. (*D*) Expression levels of top transition genes with cells ordered along the most probable transition trajectories. Solid lines, smoothed expression curves for each gene in the transition trajectory. (*E*) Dimensionality reduction of the data using QuanTC coloring for top marker genes and transition genes. Every dot represents a single cell shaped by its real time and the color scale represents the normalized expression of the respective genes.

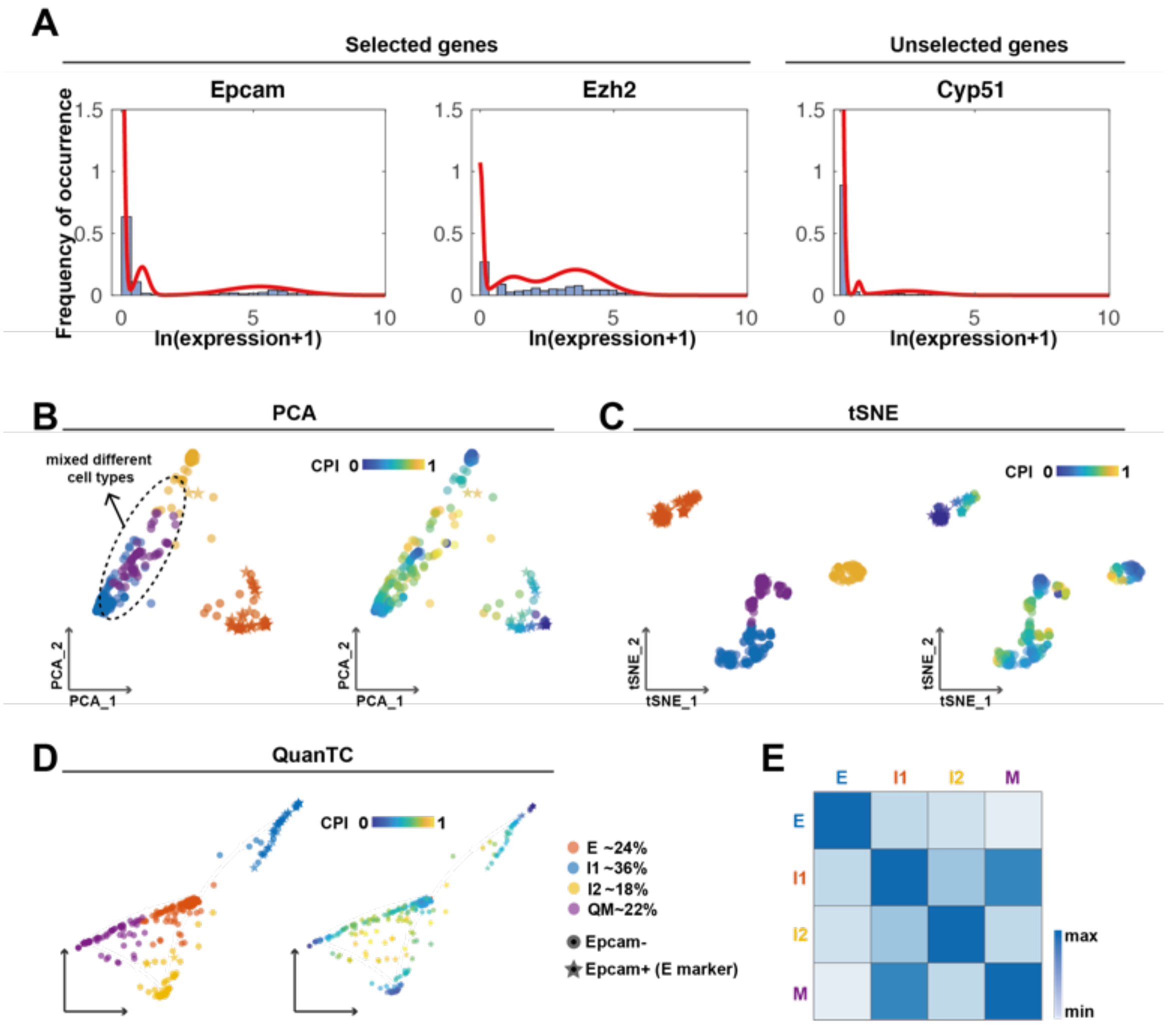

Fig. S8. Analyzing SCC dataset using QuanTC. (*A*) Histograms of the expression levels of selected informative genes and unselected genes. The red curve represents the fitted Gaussian mixture model. (*B-D*) Dimensionality reduction of the scRNA-seq data using PCA, tSNE and PRE visualization. Every dot represents a single cell colored by the clustering inferred by QuanTC (left) and CPI value (right) and its shape represents the FACS sorting criteria (Epcam$^+$ or Epcam$^-$). The percentage for each cell type is the percentage of a given cell type over the entire cell population size. (*E*) Similarities between states with color showing the value of similarities.

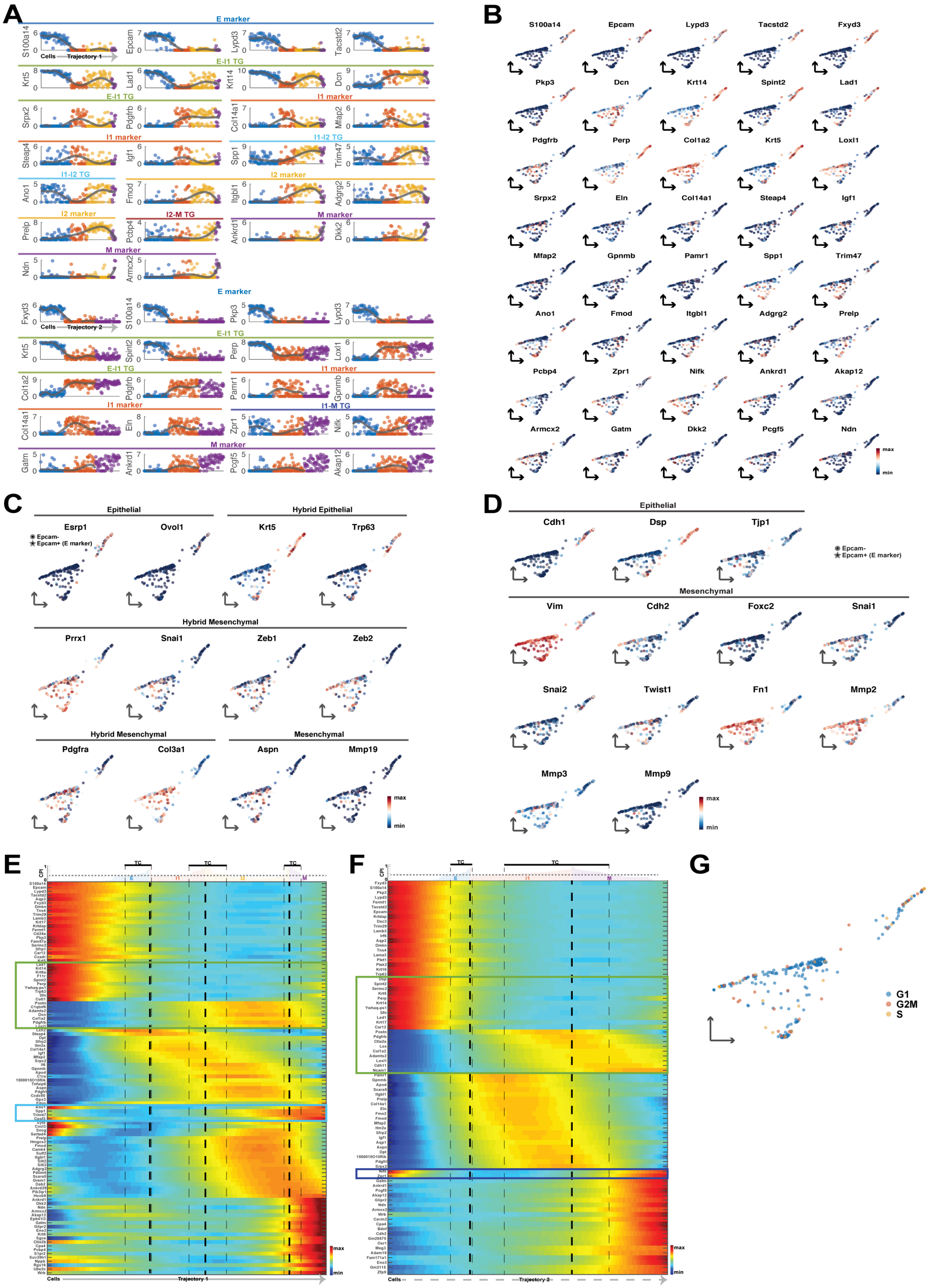

Fig. S9. Expression levels of top transition genes and marker genes involved in two main transition trajectories. (*A*) Expression levels of top transition genes and marker genes with cells ordered along transition trajectory 1 and transition trajectory 2. Solid lines, smoothed expression curves for each gene in the transition

trajectory. (*B-D*) Dimensionality reduction of the data using QuanTC coloring for top marker genes and transition genes. Every dot represents a single cell shaped by the FACS sorting criteria (Epcam$^+$ or Epcam$^-$) and the color scale represents the normalized expression of the respective genes. (*C*) Dimensionality reduction of the data using QuanTC coloring for pure epithelial genes, hybrid epithelial genes, hybrid mesenchymal genes and pure mesenchymal genes previously identified in the original study. (*D*) Dimensionality reduction of SCC data using QuanTC coloring for known epithelial genes and mesenchymal genes. (*E-F*) Heat map of normalized expression of top 20 marker genes and top 20 transition genes. Columns represent cells ordered along the transition trajectory 1 (*E*) and trajectory 2 (*F*) with rows represent genes. Coloring represents the normalized expression of each gene. Transition genes are marked in the box. Top: CPI values of each cell along the transition trajectories. (*G*) Dimensionality reduction of the data using QuanTC coloring for cell-cycle phase based on computed cell cycle scores. Every dot represents a single cell.

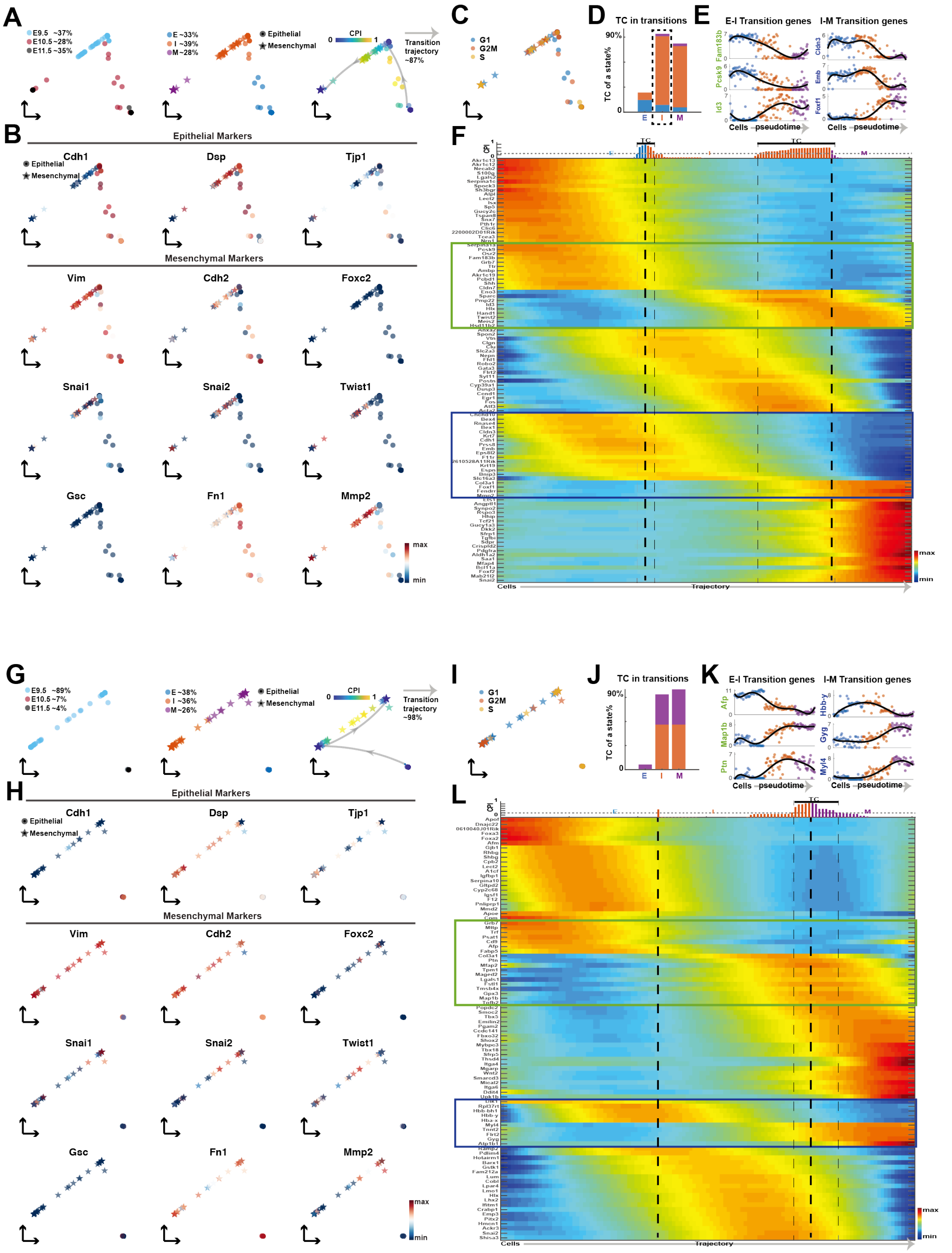

Fig. S10. Analysis of EMT during organogenesis in Intestine (top) and liver (bottom). (*A*) Visualization of cells via PRE. Each dot represents a single cell colored by the time of the tissue in mouse embryos from the original study on the corresponding dataset (left), clustering result from QuanTC (middle) and the value of CPI (right). The shape of each dot represents the cell states previously identified in the original study on the corresponding dataset. The thresholds to select TC are given in Table S1. Arrowed solid line shows the main

transition trajectory. The percentage for each cell type is the percentage of a given cell type over the entire cell population size. (*B*) Dimensionality reduction of the data coloring for known epithelial genes and mesenchymal genes. Every dot represents a single cell and its shape represents the cell states previously identified in the original study on the corresponding dataset. The color scale represents the normalized expression of the respective genes. (*C*) Dimensionality reduction of the data coloring for cell-cycle phase based on computed cell cycle scores. Every dot represents a single cell and its shape represents the cell states previously identified in the original study on the corresponding dataset. (*D*) Percentage of TC associated with each state relative to the total number of TC. (*E*) Expression levels of top transition genes with cells ordered along the most probable transition trajectory. Solid lines, smoothed expression curves for each gene in the transition trajectory. (*F*) Heat map of normalized expression of top 20 marker genes and top 20 transition genes. Columns represent cells ordered along the transition trajectory 2 and rows represent genes. Coloring represents the normalized expression of each gene. Transition genes are marked in the box. Top: CPI values of each cell along the transition trajectory. (*G*) Visualization of cells via PRE. Each dot represents a single cell colored by the time of the tissue in mouse embryos from the original study on the corresponding dataset (left), clustering result from QuanTC (middle) and the value of CPI (right). The shape of each dot represents the cell states previously identified in the original study on the corresponding dataset. The thresholds to select TC are given in Table S1. Arrowed solid line shows the main transition trajectory. (*H*) Dimensionality reduction of the data coloring for known epithelial genes and mesenchymal genes. Every dot represents a single cell and its shape represents the cell states previously identified in the original study on the corresponding dataset. The color scale represents the normalized expression of the respective genes. (*I*) Dimensionality reduction of the data coloring for cell-cycle phase based on computed cell cycle scores. Every dot represents a single cell and its shape represents the cell states previously identified in the original study on the corresponding dataset. (*J*) Percentage of TC associated with each state relative to the total number of TC. (*K*) Expression levels of top transition genes with cells ordered along the most probable transition trajectory. Solid lines, smoothed expression curves for each gene in the transition trajectory. (*L*) Heat map of normalized expression of top 20 marker genes and top 20 transition genes. Columns represent cells ordered along the transition trajectory 2 and rows represent genes. Coloring represents the normalized expression of each gene. Transition genes are marked in the box. Top: CPI values of each cell along the transition trajectory.

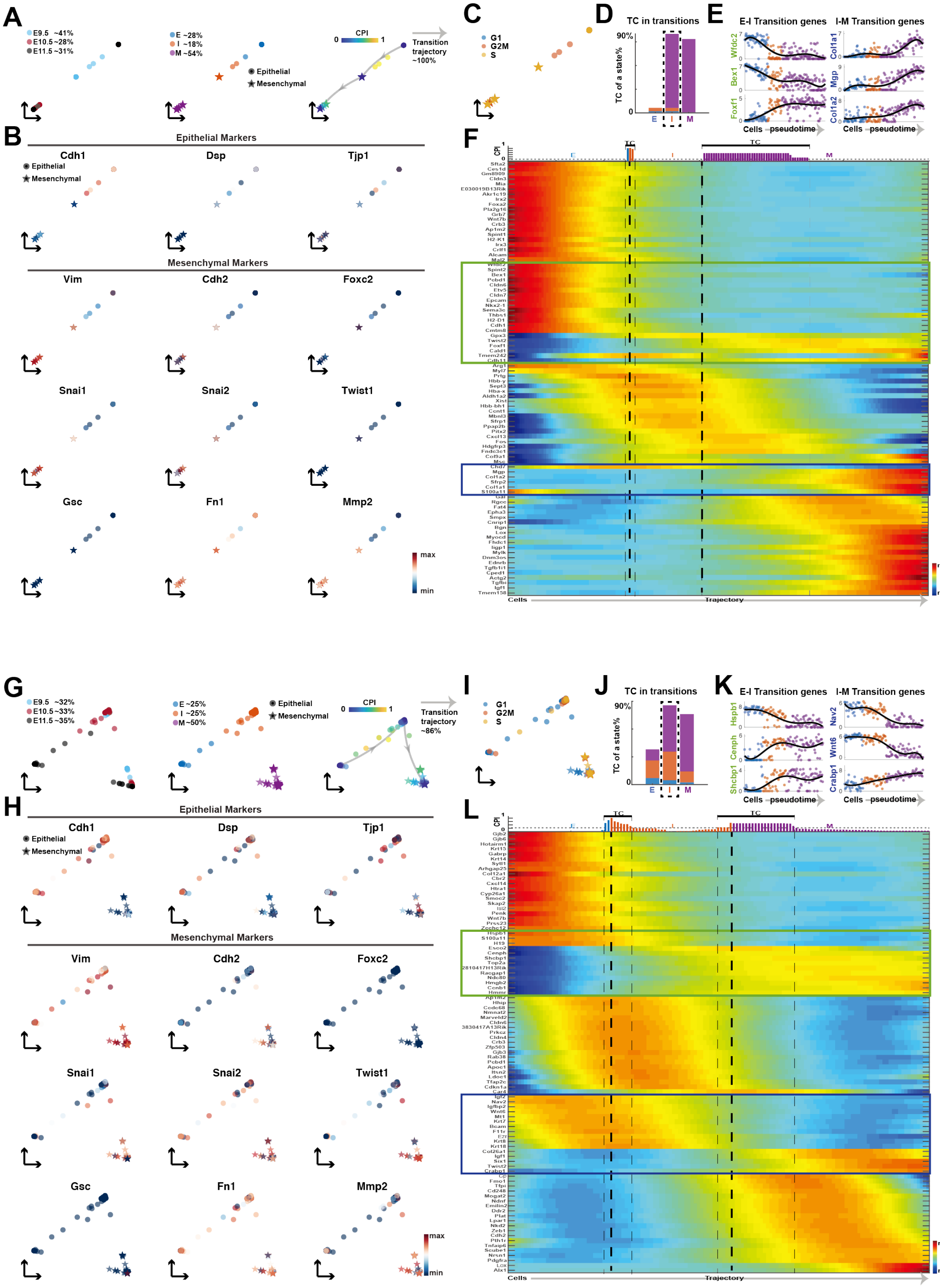

Fig. S11. Analysis of EMT during organogenesis in lung (top) and skin (bottom). (*A*) Visualization of cells via PRE. Each dot represents a single cell colored by the time of the tissue in mouse embryos from the original study on the corresponding dataset (left), clustering result from QuanTC (middle) and the value of CPI (right). The shape of each dot represents the cell states previously identified in the original study on the corresponding dataset. The thresholds to select TC are given in Table S1. Arrowed solid line shows the main transition

trajectory. The percentage for each cell type is the percentage of a given cell type over the entire cell population size. (*B*) Dimensionality reduction of the data coloring for known epithelial genes and mesenchymal genes. Every dot represents a single cell and its shape represents the cell states previously identified in the original study on the corresponding dataset. The color scale represents the normalized expression of the respective genes. (*C*) Dimensionality reduction of the data coloring for cell-cycle phase based on computed cell cycle scores. Every dot represents a single cell and its shape represents the cell states previously identified in the original study on the corresponding dataset. (*D*) Percentage of TC associated each state relative to the total number of TC. (*E*) Expression levels of top transition genes with cells ordered along the most probable transition trajectory. Solid lines, smoothed expression curves for each gene in the transition trajectory. (*F*) Heat map of normalized expression of top 20 marker genes and top 20 transition genes. Columns represent cells ordered along the transition trajectory 2 and rows represent genes. Coloring represents the normalized expression of each gene. Transition genes are marked in the box. Top: CPI values of each cell along the transition trajectory. (*G*) Visualization of cells via PRE. Each dot represents a single cell colored by the time of the tissue in mouse embryos from the original study on the corresponding dataset (left), clustering result from QuanTC (middle) and the value of CPI (right). The shape of each dot represents the cell states previously identified in the original study on the corresponding dataset. The thresholds to select TC are given in Table S1. Arrowed solid line shows the main transition trajectory. (*H*) Dimensionality reduction of the data coloring for known epithelial genes and mesenchymal genes. Every dot represents a single cell and its shape represents the cell states previously identified in the original study on the corresponding dataset. The color scale represents the normalized expression of the respective genes. (*I*) Dimensionality reduction of the data coloring for cell-cycle phase based on computed cell cycle scores. Every dot represents a single cell and its shape represents the cell states previously identified in the original study on the corresponding dataset. (*J*) Percentage of TC associated with each state relative to the total number of TC. (*K*) Expression levels of top transition genes with cells ordered along the most probable transition trajectory. Solid lines, smoothed expression curves for each gene in the transition trajectory. (*L*) Heat map of normalized expression of top 20 marker genes and top 20 transition genes. Columns represent cells ordered along the transition trajectory 2 and rows represent genes. Coloring represents the normalized expression of each gene. Transition genes are marked in the box. Top: CPI values of each cell along the transition trajectory.

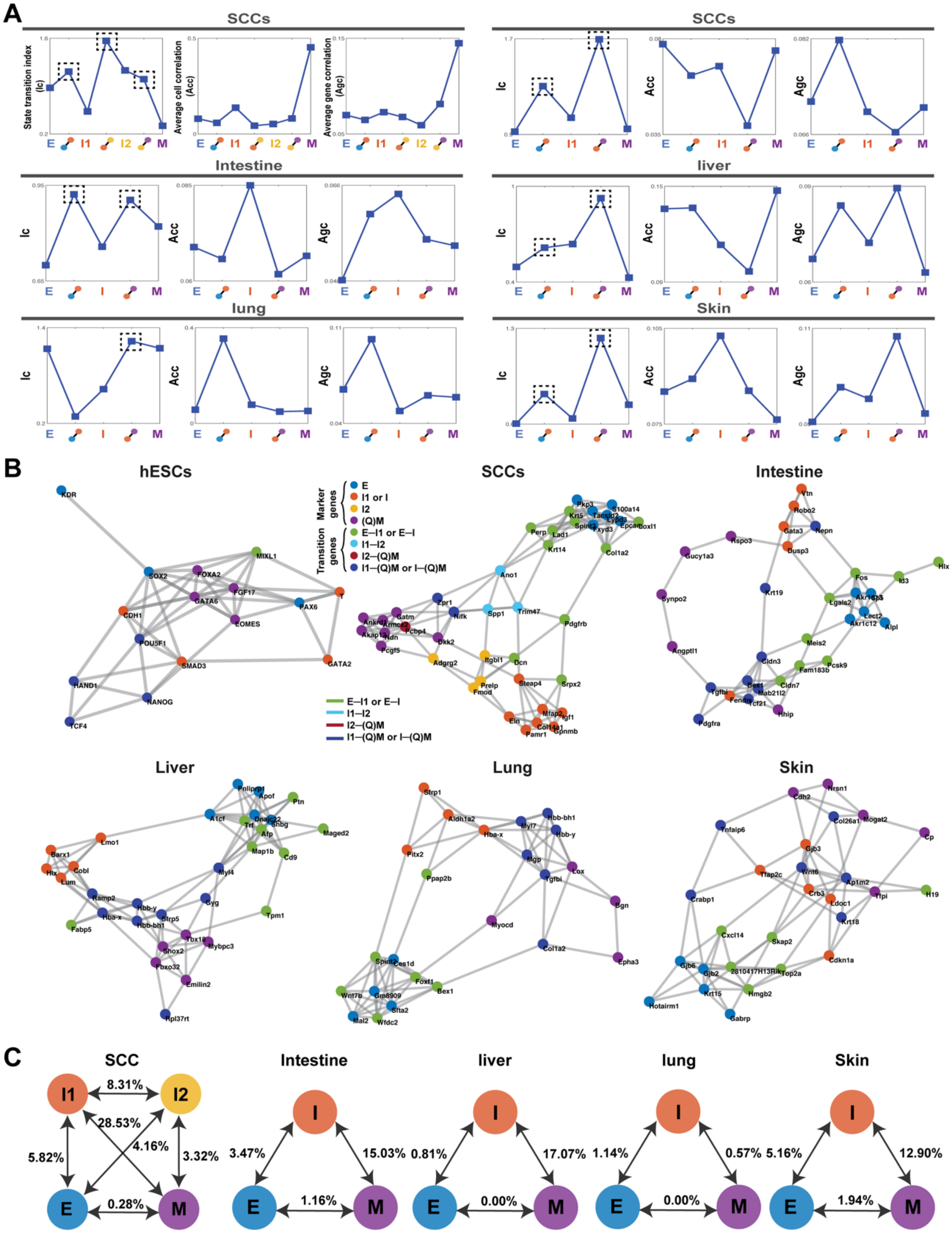

Fig. S12. State transition index and gene regulatory networks for the EMT datasets and their comparisons with QuanTC outputs. (*A*) State transition index of relatively stable cells in each state and the TC between states. Dashed box: TC with high value of state transition index. (*B*) Gene regulatory networks of top marker genes and transition genes using the PIDC algorithm from the datasets (the top ~80% of edges are shown). The thresholds to select top genes and edges are given in Supplementary Table. S1. Each dot represents a gene colored by its type. Graph edges indicate the top interactions and the length of the edge is inversely proportional to the interaction strength between genes. (*C*) EMT cell lineage inferred from datasets, with

node colors consistent with previous figures. The arrow represents potential transition between states, and number represents the percentage of TC among total number of cells.

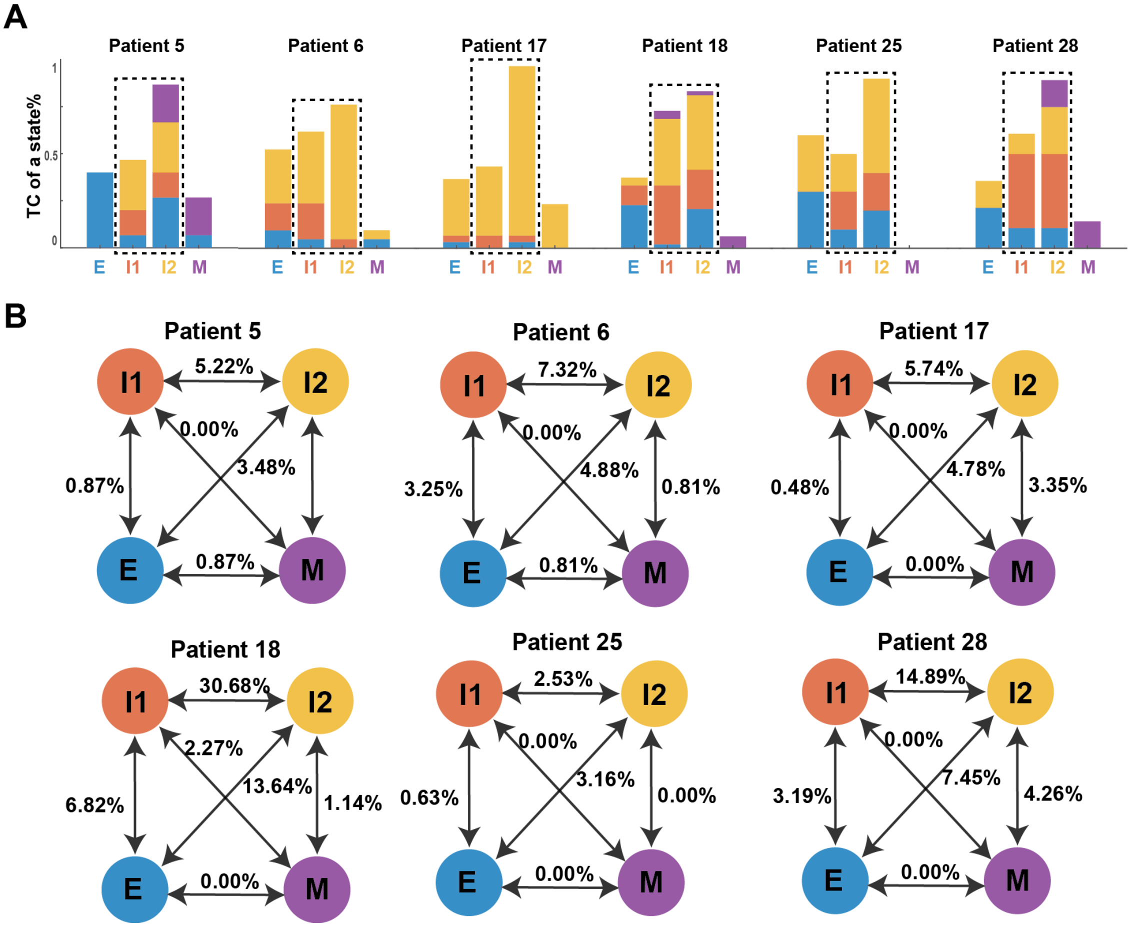

Fig. S13. (*A*) Percentage of TC in each state relative to the total number of TC from the six patients in HNSCC dataset. (*B*) EMT cell lineage inferred from datasets, with node colors consistent with previous figures. The arrow represents potential transition between states, and number represents the percentage of TC among total number of cells.

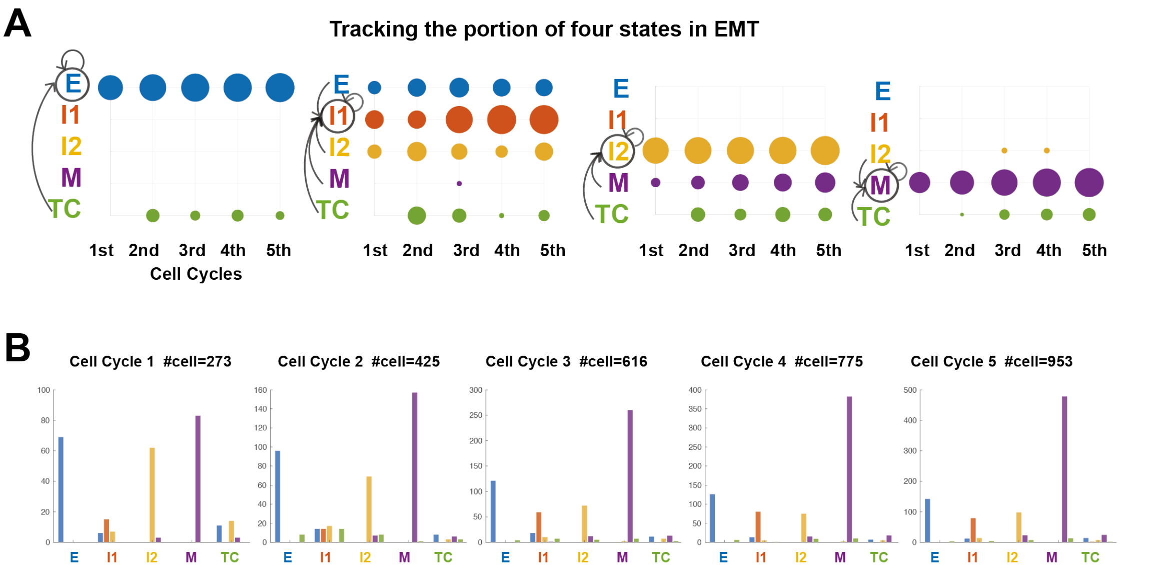

Fig. S14. Simulation of the model when I1 state is non-proliferative. (*A*) A simulation dataset: the proportion of each state induced by the previous cell states at the end of each cell cycle. The size of the dot is proportional to the number of cells, and the color denotes the cell states of the mother cell. The arrows represent the occurred state transitions and the circle represents the state of the daughter cell. (*B*) Histogram of the number of cell population at the end of each cycle. The color denotes the mother cell states. The x-labels represent the states of the daughter cell.

Supplementary Tables

Table S1. Thresholds of CPI values and to select top genes and edges in PIDC

	hESC	SCC	Intestine	Liver	Lung	Skin
Number top marker genes	4	4	5	5	4	5
Number top transition genes	4	6	8	8	6	6
Percentage of top edges	69%	84%	86%	80%	74%	80%
Thresholds of CPI to select TC	0.34	0.34	0.34	0.2	0.1	0.2
